# A critical assessment of the abuse, dependence and associated safety risks of naturally occurring and synthetic cannabinoids

**DOI:** 10.3389/fpsyt.2024.1322434

**Published:** 2024-06-10

**Authors:** David J. Heal, Jane Gosden, Sharon L. Smith

**Affiliations:** ^1^ DevelRx Limited, Nottingham, United Kingdom; ^2^ Department of Life Sciences, University of Bath, Bath, United Kingdom

**Keywords:** delta 9 tetrahydrocannabinol, THC - tetrahydrocannabinol, cannabidiol (CBD), cannabis, CB1 - CB2 cannabinoid receptors, abuse, dependence

## Abstract

Various countries and US States have legalized cannabis, and the use of the psychoactive^1^ and non-psychoactive cannabinoids is steadily increasing. In this review, we have collated evidence from published non-clinical and clinical sources to evaluate the abuse, dependence and associated safety risks of the individual cannabinoids present in cannabis. As context, we also evaluated various synthetic cannabinoids. The evidence shows that delta-9 tetrahydrocannabinol (Δ^9^-THC) and other psychoactive cannabinoids in cannabis have moderate reinforcing effects. Although they rapidly induce pharmacological tolerance, the withdrawal syndrome produced by the psychoactive cannabinoids in cannabis is of moderate severity and lasts from 2 to 6 days. The evidence overwhelmingly shows that non-psychoactive cannabinoids do not produce intoxicating, cognitive or rewarding properties in humans. There has been much speculation whether cannabidiol (CBD) influences the psychoactive and potentially harmful effects of Δ^9^-THC. Although most non-clinical and clinical investigations have shown that CBD does not attenuate the CNS effects of Δ^9^-THC or synthetic psychoactive cannabinoids, there is sufficient uncertainty to warrant further research. Based on the analysis, our assessment is cannabis has moderate levels of abuse and dependence risk. While the risks and harms are substantially lower than those posed by many illegal and legal substances of abuse, including tobacco and alcohol, they are far from negligible. In contrast, potent synthetic cannabinoid (CB1/CB2) receptor agonists are more reinforcing and highly intoxicating and pose a substantial risk for abuse and harm. ^1^ “Psychoactive” is defined as a substance that when taken or administered affects mental processes, e.g., perception, consciousness, cognition or mood and emotions.

## Introduction

The psychoactive (definition: exerting effects on mental processes, e.g. perception, consciousness, cognition or mood and emotions) and medicinal properties of the cannabis plant species, *Cannabis sativa* and *Cannabis indica*, have been known for thousands of years. The first written reports of their medical and recreational use were in Asia, occurring in the third millennium B.C. and evidence exists to suggest that the first human use of these plants dates as far back as the Neolithic period several thousand years earlier.

**Figure 1:**
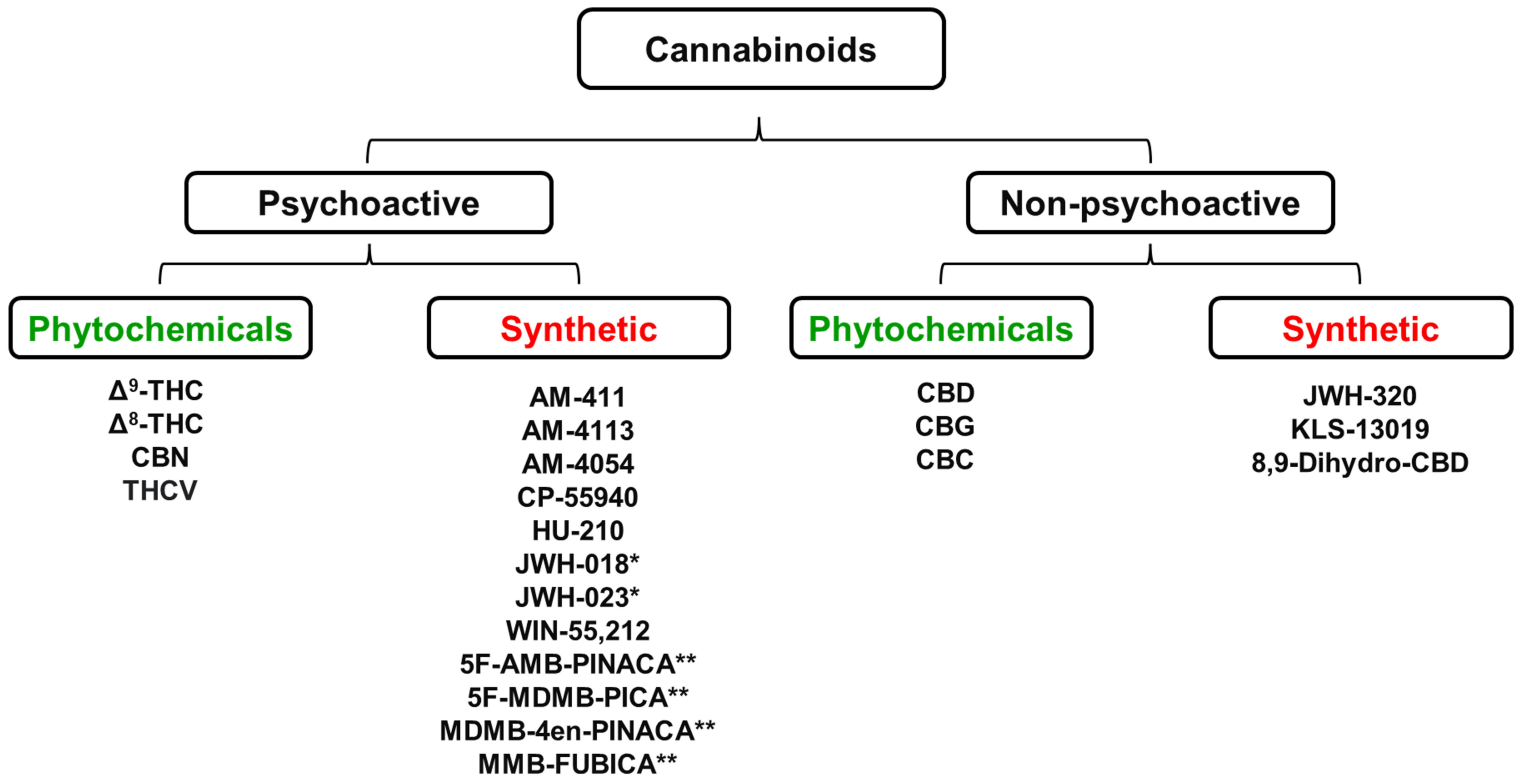
Some of the most abundant cannabinoids present in the *Cannabis sativa* plant together with a range of chemically synthesized analogues of these naturally occurring cannabinoids * Denotes “first generation” synthetic psychoactive cannabinoids that have been detected in samples of the illegal substances “Spice” and “K2”. ** Denotes “second generation” synthetic psychoactive cannabinoids that have been detected in samples of the illegal substance called “Incense”. CBC, Cannabichromene; CBD, Cannabidiol; CBG, Cannabigerol; CBN, Cannabinol; Δ^9^-THC, Δ^9^-Tetrahydrocannabinol; Δ^8^-THC, Δ^8^-Tetrahydrocannabinol; THCV, Tetrahydrocannabivarin. Figure created by the authors.

Cannabis plants produce more than 400 chemical compounds and approximately 60 of them are biologically active. The two best known plant-derived cannabinoids (phytocannabinoids) are Δ^9^-tetrahydrocannabinol (Δ^9^-THC), which is the predominant psychoactive molecule present in cannabis, and cannabidiol (CBD) which is non-psychoactive. These compounds together with a range of other plant-derived and synthetic cannabinoids that are discussed in this review are shown in **Figure 1**.

Biologically active cannabinoids can be subdivided into two major pharmacological classes, i.e., “psychoactive” and “non-psychoactive”. Psychoactive cannabinoids affect mental processes, e.g., perception, consciousness, cognition, mood, or emotions. Psychoactive cannabinoids are also categorized as intoxicating because they impair reflexes, decrease cognitive function, and produce a psychological state ranging from euphoria to stupor that is accompanied by loss of inhibitions and control. It is important to emphasize that describing a cannabinoid as “non-psychoactive” does not imply the compound is devoid of pharmacological effects in the brain. CBD is the most abundant, non-psychoactive cannabinoid produced by the cannabis plant. CBD has powerful anticonvulsant actions that derive from its ability to reduce neuronal excitability by antagonism of G protein-coupled receptor-55 (GPR55) receptors, desensitization of transient receptor potential vanilloid-1 (TRPV1) receptors, and inhibition of adenosine transport by blockade of the equilibrative nucleoside transporter 1 (ENT-1) (1). CBD under the trade name of Epidiolex™ (highly purified CBD extracted from cannabis plants) has been tested and clinically approved to prevent seizures associated with Lennox-Gastaut and Dravet syndromes, which are two rare forms of childhood epilepsy (2). The beneficial pharmacological properties of other cannabinoids have also been harnessed as prescription medicines; they include Marinol™ (dronabinol; chemically synthesized Δ^9^-THC) (3), Syndros™ (dronabinol) (4), Cesamet™ (nabilone, a synthetic chemical analogue of Δ^9^-THC) (5), and Sativex^®^ (nabiximols; a 1:1 mixture of plant-derived Δ^9^-THC and CBD) (6).

A detailed history of legal restrictions on the use of cannabis is beyond the scope of this review, but one of the major initiatives that resulted in strict control over the production, distribution and sale of cannabis was the 1961 United Nations Conference for Adoption of a Single Convention on Narcotic Drugs. In many countries, the unlicensed cultivation of cannabis plants is illegal, as is the possession and distribution of the plants or their chemical products. The US Controlled Substances Act, 1973, and its UK equivalent, the Home Office Misuse of Drugs Act, 1971, classify cannabis and its biologically active products as illegal drugs that are “dangerous or otherwise harmful”. Cannabis and its psychoactive phytocannabinoids constituents have no approved medical use and, therefore, reside in Schedule 1 (C-I) according to the US Controlled Substances Act and the UK Home Office Misuse of Drugs Act. However, specific products containing Δ^9^-THC or close analogues with an approved medical use have been placed in less restrictive controlled drug schedules, i.e. Marinol™ in C-III, Syndros™ in C-II, Cesamet™ in C-II and Sativex^®^ in C-IV. This has created the confusing situation whereby Δ^9^-THC sits in 4 separate controlled drug schedules depending on the specific product and medical use, or lack of it. After many decades of unsuccessfully attempting to eradicate the illegal use of cannabis by rigorous law enforcement often backed up by harsh penalties for drug possession, some countries and a majority of States in the USA have taken the significant step of decriminalizing or legalizing the use of cannabis for medical and/or recreational purposes. The Netherlands was the first country to legalize the recreational use of cannabis in 1976, and subsequently, it has been joined by 6 others, i.e., Argentina, Belgium, Canada, Germany, Italy and Thailand. In the USA, cannabis is still illegal at the federal level, but it has been legalized for recreational use by adults, aged 21 years and older, by Washington, D.C. and 23 other States. Legalized medical use programs for cannabis have been instituted in 38 US States and many other countries around the world including the UK. Although CBD is not psychoactive, CBD deriving from hemp was only legalized in 2018, and in a few USA States where it has not been removed from the local controlled substances acts, e.g., Idaho and Nebraska, its use is still illegal. Buying and using CBD in Europe is a mixed picture with no restrictions in some countries, e.g., UK, France, Germany, Italy, Netherlands, Norway, Spain and Sweden, but its use a legal “grey area” in some countries, and it is illegal in others, e.g., Albania, Denmark, Finland, Iceland, Ireland, Portugal, Romania, and Russia. Clearly, the political landscape for the legal regulation of cannabis is changing with some countries/States moving towards liberalization, while others are holding a firm conservative line on the *status quo*. The move towards the legalization of cannabis and cannabinoids for recreational and self-prescribed medical use has shifted to the point where an objective assessment of their abuse and dependence risks is warranted. This information can then be used to make predictions about their impact on personal and public safety in the situation where society has unrestricted access to these substances.

When attempting to assess objectively the abuse and dependence risks posed by cannabis and cannabinoid chemicals, it is important to factor-in the increasing psychoactive potency of the material that is being used. A global trend for selective breeding in the cultivation of cannabis plants has resulted in a dramatic increase in the concentration of Δ^9^-THC in present marijuana, cannabis resin and hash oil samples (7–10).

A second factor to be considered in this analysis is the impact of legalization on the level of problem cannabis use. It has been estimated that almost 200 million people around the world engage in the use of cannabis (11). Although the estimates vary across studies and age groups, one inescapable consequence of cannabis legalization is a substantial increase in the number of cannabis users (12–14). In those US States where cannabis use has been legalized, there has also been a steep increase in the incidence and prevalence of cannabis use disorder (C.U.D.) (12–17). Once again, the numbers vary between studies, but a consistent theme is the incidence of C.U.D. is increasing across all sections of cannabis using population, i.e. not only those taking the drug for recreational purposes, but also amongst people who using the drug to self-medicate psychological and physical conditions (13, 16–18).

Other investigators have adopted a holistic approach to assessing the risks and harms of cannabis to the user and society, and bench-marked them relative to other illicit and legalized substances of abuse, including tobacco and alcohol (19–21). These analyses have consistently concluded that the risks posed by cannabis were moderate, and substantially lower than the harms not only of many illegal drugs, e.g., opiates and stimulants, but also the legal drugs, tobacco and alcohol.

In this review, we have adopted the scientific methodology that is employed to evaluate the abuse and dependence risks posed by novel CNS-active drugs being developed for medical or veterinary use. When drugs are assessed for medical use, a “benefit/risk” analysis is performed in which unmet medical need and clinical efficacy are balanced against the drug’s potential harms in terms of tolerability, safety, and abuse/dependence risks. To illustrate how this process works, a range of hypothetical scenarios and approval decisions are shown in **Table 1**. Recreational use of drugs offers no tangible health benefits to the user, and consequently, the acceptable risks for recreational use should be set at a lower level. Self-medication to treat diseases/disorders that are either self-diagnosed or unresponsive to medical treatment fall in the middle where the perceived health benefits would justify a higher level of safety risk.

**Table 1:** Benefit risk analysis applied to the use of cannabinoids for medical and recreational use.

Purpose	Benefit	Risk	Benefit/risk analysis
Prescription clinical use			
High unmet clinical need	High level of medical value. Potentially life-saving intervention.	Substantial risk in terms of patient tolerability and safety.	Positive
Low unmet clinical need	Moderate benefit over existing safe and effective drugs.	Low risk in terms of tolerability and safety.	Positive
Low unmet clinical need	Small benefit over existing safe and effective drugs.	Substantial risk in terms of patient tolerability and safety.	Negative
Recreational use			
No tangible health benefits	Positive reward. Pleasurable subjective effects	No risk in terms of tolerability and safety.	Positive
No tangible health benefits	Positive reward. Pleasurable subjective effects.	Low risk in terms of tolerability and safety.	Positive
No tangible health benefits	Positive reward. Pleasurable subjective effects.	Moderate risk in terms of tolerability and safety. Lower risk than alcohol and tobacco.	Possible
No tangible health benefits	Positive reward. Pleasurable subjective effects.	Substantial risk in terms of tolerability and safety.	Negative

We have applied this methodology to examples of psychoactive and non-psychoactive cannabinoids. The findings have been supplemented by evidence obtained from research on a range of synthetic cannabinoids, including some that constitute the active components in the potent illicit compounds, “Spice”, “K2” and “Incense”. The primary evidence was selected from well controlled, peer-reviewed, scientific and clinical studies published in reputable international scientific journals. Findings that have been replicated in multiple laboratories were considered to have higher evidential standing. Because the abuse and dependence risks of individual cannabinoids were analyzed, greater weight was placed on results generated using pharmacologically pure compounds in placebo- or vehicle-controlled studies. Observations from self-reported sources and those involving cannabis, which comprises multiple cannabinoids usually supplemented with tobacco when smoked, were assigned lower weighting. Finally, because there has been a great deal of interest and speculation about the potential of CBD to counteract and mitigate the abuse and dependence potential of Δ^9^-THC, we have evaluated the published non-clinical and clinical research to determine whether these claims have any evidential basis.

## Pharmacology

The endogenous cannabinoids (endocannabinoids) produced by humans and other mammalian species signal through two receptor subtypes, i.e., type 1 (CB1) and type 2 (CB2). The CB1 receptor was first discovered in the mammalian brain by Devane et al. (1988) (22). Matsuda et al. (1990) (23) determined its DNA sequence and demonstrated that various psychoactive botanical and synthetic cannabinoids including Δ^9^-THC and nabilone were agonists at this receptor. Evidence identifying the CB1 receptor as the mediator for the psychoactive effects of these cannabinoids was provided by the observation that CBD which is not psychoactive showed minimal agonist activity. Subsequent anatomical mapping studies revealed that CB1 receptors are widely distributed in the mammalian brain; in fact, the CB1 receptor is the most widely-expressed receptor protein from the GPCR family in the CNS (23, 24). CB1 receptors are present in the cortical and limbic areas, e.g., cerebral cortex, septum, amygdala, hippocampus, subcortical regions, e.g. basal ganglia, and cerebellum and hypothalamus, and the dorsal horn of the spinal cord (24). The ubiquitous distribution of CB1 receptors explains why the CNS effects of the psychoactive cannabinoids are diverse and profound. This receptor is also widely distributed in peripheral tissues including skeletal muscle, liver and pancreatic islet cells (24).

The CB2 receptor, which is predominantly located in peripheral tissues, was identified, sequenced, and cloned by Munro et al. (1993) (25). This cannabinoid receptor mediates many of the peripheral actions of the cannabinoids including analgesia, anti-inflammatory, immunosuppressant, and antiemetic effects. From a translational validity perspective, it is important to note that the CB2 receptor with approximately 80% amino acid sequence homology between humans and rodents exhibits greater species differences between humans and rodents than the CB1 receptor (26, 27). Although there are low expression levels of the CB2 receptor in various brain regions, the consensus view is the psychoactive effects of the cannabinoids do not rely on activation of this receptor subtype.

The magnitude of the pharmacological effect of a compound is determined by 3 factors: (i) affinity for the molecular target, (ii) intrinsic activity, and (iii) concentration at the target site. The affinities for the CB1 receptor of a range of naturally occurring and synthetic psychoactive and non-psychoactive cannabinoids is reported in **Table 2**. The table is not intended to list every compound or every published report of CB1 and CB2 receptor affinities; rather it is intended to be sufficiently comprehensive to illustrate the different categories and the spread of Ki values that have been reported for many of these compounds. Ki values in the 10-100nM range are generally considered to signify moderate receptor affinity. The results show that Δ^9^-THC and the other naturally occurring psychoactive cannabinoids, i.e., Δ^8^-THC, cannabinol (CBN) and Δ9-Tetrahydrocannabivarin (THCV), are moderately potent CB1 ligands and with similar Ki values for CB1 and CB2 receptors, these compounds are also non-selective. In contrast, CBD and other the non-psychoactive compounds, with the possible exception of cannabichromine (CBC), have weak to negligible affinity for either CB receptor. Nabilone has 5-10x higher affinity for the CB1 receptor than Δ^9^-THC ligand, but similarly has no CB1/CB2 receptor selectivity. The “first generation” synthetic psychoactive cannabinoids that have been used in “Spice/K2”, e.g., JWH-018 and JWH-023, have higher affinity for CB1 and CB2 receptors than the psychoactive cannabinoids in cannabis. However, many of the “second generation” synthetic compounds employed in illegal products like “Incense”, e.g., 5F-EDMB-PINACA, MDMB-4en-PINACA, and FUB-AKB48, have nanomolar CB1/CB2 affinities making them extremely potent cannabinoid receptor ligands.

**Table 2:** CB1 and CB2 receptor affinities of psychoactive and non-psychoactive cannabinoids.

Compound	CB1 receptor Ki = nM	CB2 receptor Ki = nM	Source
Psychoactive phytocannabinoids			
Δ^9^-THC ^A^	25.1	35.2	(28)
Δ^9^-THC	36	31	(29)
Δ^9^-THC	18	42	(30)
Δ^9^-THC	40.7	36.4	(31)
Δ^9^-THC	35.6	8.5	(32)
Δ^9^-THC	7.5	6.5	(33)
Δ^9^-THC	8.1	7.5	(34)
Δ^9^-THC	ND	14.8	(35)
Δ^9^-THC	53.3	75.3	(36)
Δ^8^-THC	78	12	(30)
Δ^8^-THC	6.6	6.4	(33)
CBN ^A^	239	440	(28)
CBN	12.7	16.4	(32)
CBN	75	73	(30)
CBN	308	96	(31)
THCV	22	47	(29)
THCV	75.4	62.8	(37)
THCV	22	105	(30)
THCV	46.6	ND	(38)
Non-psychoactive phytocannabinoids			
CBD ^A^	ND	2860	(28)
CBD	200	240	(29)
CBD	1458	372	(32)
CBD	151	4582	(30)
CBD	>10,000	ND	(39)
CBD	>10,000	>10,000	(40)
CBC	11	27	(29)
CBC	714	257	(32)
CBG	1300	490	(29)
CBG	897	153	(32)
CBG	3090	2919	(30)
CBDV	>10,000	140	(29)
CBDV	14711	574	(32)
CBDV	503	3970	(30)
CBV	565	4780	(30)
Psychoactive synthetic cannabinoids			
Nabilone	1.84	2.19	(41)
5F-EDMB-PINACA ^C^	0.378	0.442	(34)
5F-MMB-PICA ^C^	6.5	3.4	(34)
AM251	3.43	124	(42)
AM-2201	1.0	2.6	(43)
AM4113	0.89	92	(42)
AM-8936	0.55	ND	(44)
APP-BINACA ^C^	1.07	9.34	(34)
CP 47,497	7.21	7.04	(33)
CP 55,940	13	29	(29)
CP 55,940	8.18	7.95	(33)
CP 55,940	0.997	0.618	(34)
Cp 55,940	3.72	2.55	(36)
CP 55,940	ND	0.4	(35)
FUB-144 ^C^	27.2	1.19	(34)
FUB-AKB48 ^C^	1.30	0.823	(34)
HU-210 ^A^	0.25	0.4	(28)
HU-210	8.76	8.84	(33)
HU-210	0.061	0.524	(36)
JWH-018 ^B^	9.0	2.9	(45)
JWH-018 ^B^	9.0	2.9	(43)
JWH-018 ^B^	ND	5.6	(35)
JWH-073 ^B^	9.0	27	(43)
JWH-073 ^B^	8.9	38	(46)
JWH-073 ^B^	ND	9.8	(35)
JWH-210	0.46	0.49	(43)
JWH-210	0.46	0.69	(46)
MDMB-4en-PINACA ^C^	1.40	0.213	(34)
MMB-4en-PICA ^C^	39	15.6	(34)
MMB-FUBICA ^C^	12	22.5	(34)
WIN 55,212	8.08	3.22	(47)
WIN 55,212	6.89	7.78	(33)
WIN 55,212	3.72	2.55	(36)
Non-psychoactive synthetic cannabinoids			
JWH-320	>10,000	ND	(46)
KLS-13019	>10,000	>10,000	(39)

A = Meta-analysis of affinities for human CB1 and CB2 receptors.

B = First generation synthetic psychoactive cannabinoids found in “Spice” and “K2.”

C = Second generation synthetic psychoactive cannabinoids found in “Incense.”

CBC, cannabichromene; CBD, cannabidiol; CBG, cannabigerol; CBDV, cannabidivarin; CBN, cannabinol; CBV, cannabivarin.

Δ^8^-THC, (-)Δ^8^-tetrahydrocannabinol; Δ^9^-THC, (-)Δ^9^-tetrahydrocannabinol;

THCV, Δ^9^-tetrahydrocannabivarin.

ND, not determined.

Yellow shading indicates replicated data for individual compounds.

The functional actions of the cannabinoids are reported in **Table 3**. The psychoactive cannabinoids in cannabis are all partial agonists of CB1 and CB2 receptors. This point is emphasized when the Ki and EC_50_ values are compared. Although 50% receptor occupancy is achieved at 10-50 nM, the concentration to elicit 50% agonist efficacy is generally 5-10x higher. The findings also show that CBD and the other naturally occurring, non-psychoactive cannabinoids have no pharmacologically relevant CB1 receptor agonist activity. JWH-018 functions as a full agonist at the CB1 receptor, and probably so does JWH-023. “Second generation” synthetic psychoactive cannabinoids, e.g., 5F-EDMB-PINACA, MDMB-4en-PINACA, and FUB-AKB48, not only have nanomolar affinity for the CB1 receptor, they are also full agonists with EC_50_ values in this range making their effects hundreds of times more powerful than compounds like Δ^9^-THC.

**Table 3:** Pharmacological profiles of various psychoactive and non-psychoactive cannabinoids.

Compound	CB1 receptor			CB2 receptor			Source
	Ki = nM	EC_50_ = nM	E_max_ (%)	Ki = nM	EC_50_ = nM	E_max_ (%)	
Psychoactive phytocannabinoids							
Δ^9^-THC	36	240	56	31	18.0	76	(29)
Δ^9^-THC	7.49	245	48	6.51	2.34	-27	(33)
Δ^9^-THC	18	269	ND	42	327	ND	(30)
Δ^9^-THC	8.09	89.9	128	7.5	20.3	46.5	(34)
Δ^9^-THC	53.3	16.5	ND	75.3	41.8	ND	(36)
Δ^9^-THC	ND	13.5	38%	ND	ND	ND	(23)
Δ^9^-THC	ND	ND	ND	14.8	57.9	49.2	(35)
Δ^9^-THC (*m*)	ND	24.3	120	ND	ND	ND	(48)
Δ^8^-THC	6.60	117	41	6.38	1.32	-16	(33)
Δ^8^-THC	ND	27.4	36	ND	ND	ND	(23)
CBN	75	307	ND	73	289	ND	(30)
CBN	1130	>1,000	ND	301	>1,000	ND	(36)
THCV	22	260	59	47	280	79	(29)
Non-psychoactive phytocannabinoids							
CBD	200	>10,000	26	240	>10,000	61	(29)
CBD	151	1469	ND	4582	104	ND	(30)
CBC	11	190	68	27	7.1	76	(29)
CBG	1300	120	68	490	130	39	(29)
CBG	3090	>10,000	ND	2919	1158	ND	(30)
CBDV	>10,000	>10,000	68	140	5.0	51	(29)
CBDV	503	>10,000	ND	3970	>10,000	3.0	(30)
CBV	565	>10,000	ND	4780	>10,000	ND	(30)
Psychoactive synthetic cannabinoids							
Nabilone	ND	16.6	60	ND	ND	ND	(23)
5F-EDMB-PINACA ^B^	0.378	1.60	381	0.442	0.442	161	(34)
5F-MMB-PICA ^B^	6.45	5.07	295	3.4	1.65	77.8	(34)
AM-8936	0.55	1.4	94	ND	ND	ND	(44)
APP-BINACA ^B^	107	344	180	9.34	2.24	83.2	(34)
CP 47,497	7.21	5.75	30	7.04	0.22	49	(33)
CP47,497 (*m*)	ND	102	212	ND	ND	ND	(48)
CP 55,940	13	7.7	100	29	4.0	100	(29)
CP 55,940	8.18	1.00	60	7.95	3.89	57	(33)
CP 55,940	0.797	4.19	335	0.618	0.715	126	(34)
CP 55,940	3.8	5.5	72	4.2	ND	ND	(49)
CP 55,940	ND	1.5	100	ND	ND	ND	(44)
CP 55,940	ND	ND	ND	0.4	3.3	67.3	(35)
CP 55,940	3.72	1.83	ND	2.55	2.89	ND	(36)
CP 55,940 (*r*)	0.11	0.47	165	ND	ND	ND	(50)
CP 55,940 (*m*)	ND	4.1	198.7	ND	ND	ND	(48)
FUB-144 ^B^	27.2	90.6	218	1.19	4.61	194	(34)
FUB-AKB48 ^B^	1.30	6.94	368	0.823	0.424	131	(34)
HU-210	8.76	0.078	67	8.84	0.60	46	(33)
HU-210	0.061	0.197	ND	0.524	0.578	ND	(36)
HU-210 (*r*)	0.73	0.55	140	ND	ND	ND	(50)
JWH-018 ^A^	9.8	14.7	79	3.1	ND	ND	(49)
JWH-018 ^A^	3.38	20.2	163	ND	ND	ND	(51)
JWH-073 ^A^	ND	ND	ND	9.8	16.9	62.6	(35)
JWH-073 ^A^ (*r*)	8.9	105	29	ND	ND	ND	(50)
JWH-073 ^A^ (*m*)	ND	25.6	186	ND	ND	ND	(48)
MDMB-4en-PINACA ^B^	1.4	3.30	304	0.213	1.34	63.6	(34)
MMB-4en-PICA ^B^	39	60.7	298	15.6	12.4	77.6	(34)
MMB-FUBICA ^B^	12	59.7	278	22.5	22.1	52.1	(34)
WIN 55,212	6.89	40.7	68	7.78	5.62	32	(33)
WIN 55,212	21.7	38.9	68	2.3	ND	ND	(49)
WIN 55,212	62.3	24.0	ND	3.30	0.407	ND	(36)
WIN 55,212 (*r*)	1.89	151	156	ND	ND	ND	(50)
WIN 55,212 (*m*)	ND	39.4	203	ND	ND	ND	(48)
Non-psychoactive synthetic cannabinoids							
JWH-320	>10,000	*No data*		ND	*No data*		*No data*
KLS-13019	>10,000	*No data*		>10,000	*No data*		*No data*

A = First generation synthetic psychoactive cannabinoids found in “Spice” and “K2.”

B = Second generation synthetic psychoactive cannabinoids found in “Incense.”

CBC, cannabichromene; CBD, cannabidiol; CBG, cannabigerol; CBDV, cannabidivarin; CBN, cannabinol; CBV, cannabivarin; Δ^8^-THC, (-)Δ^8^-tetrahydrocannabinol; Δ^9^-THC, (-)Δ^9^-tetrahydrocannabinol; THCV, Δ^9^-tetrahydrocannabivarin.

(r), rat; (m), mouse; ND, not determined.

Yellow shading indicates replicated data for individual compounds.

Based on the *in vitro* results, the prediction is Δ^9^-THC and other psychoactive cannabinoids in cannabis will elicit CB1 receptor-mediated effects, but their potency and efficacy *in vivo* will reflect their pharmacology as moderate affinity partial agonists. The cannabinoid tetrad in rodents which consists of analgesia, hypothermia, hypoactivity and catalepsy is an established model for evaluating CB1 receptor agonists. Δ^9^-THC evokes all of these pharmacological effects (e.g (29, 52, 53). The reported ED_50_ values for Δ^9^-THC in the tetrad model vary considerably depending on the individual component, rodent species, and conducting laboratory, but are broadly in the range 1-50 mg/kg (48, 53, 54). Δ^8^-THC and Δ^9^-THC are reported to have similar potencies in the tetrad model (52, 53, 55). CBN decreased locomotor activity, but it did not produce the typical pattern of CB1-mediated effects in the tetrad model (55). It is CNS-active, because it decreased locomotor activity and induced catalepsy after injection into the cerebral ventricles (56). THCV has relatively weak effects in the tetrad model (29) and the non-psychoactive cannabinoids, CBD, CBC, cannabigerol (CBG) and cannabidivarin (CBDV) are inactive (29, 55, 57). JWH-073 which has been found in “Spice/K2” is approximately 10x more potent than Δ^9^-THC in the tetrad model (48, 54) and some of the first generation of synthetic CB1 receptor agonists, e.g. CP 55,940, WIN 55,212, and CP 47,497, are up to 100x more potent than Δ^9^-THC (29, 48, 52–54). Although relatively little research has been conducted on the “second generation” synthetic CB1 receptor agonists, Marusich et al. (2022) (34) has reported that several of the high potency/high efficacy compounds discussed earlier are not only >100x more potent than Δ^9^-THC, but they also have an extended duration of effect.

Together the *in vitro* and *in vivo* findings characterize the psychoactive cannabinoids present in cannabis as moderate potency CB1 receptor agonists and this pharmacology translates well into the magnitude of their CNS effects. The synthetic CB1 receptor cannabinoids produce much more profound psychoactive effects as a result of increased affinity and full agonist properties.

## Abuse/dependence evaluation

All novel drugs that produce their therapeutic effect by an action in the brain are required to undergo a systematic assessment of their potential for human abuse and risk of producing a syndrome of physical dependence on withdrawal. These investigations form part of the Safety Pharmacology evaluation that is a regulatory requirement for all drugs seeking approval for medical use in humans. Guidance on the evaluation of risks for abuse and dependence have been produced by the Committee for Medicinal Products for Human Use (CHMP) of the European Medicines Agency (EMA) (58) and the Center for Drug Evaluation and Research (CDER) of the US Food and Drug Administration (FDA) (59). The regulatory requirements for non-clinical abuse potential assessments are broadly consistent between EMA and FDA, but FDA also places great store on findings from double-blind, placebo- and active-controlled, clinical trials in drug-experienced human volunteers (59). We have previously explored the similarities and differences between the approaches adopted by EMA and FDA and their implications for abuse/dependence assessments and for an in-depth review of this topic see Calderon et al. (2015) (60).

**Figure 2:**
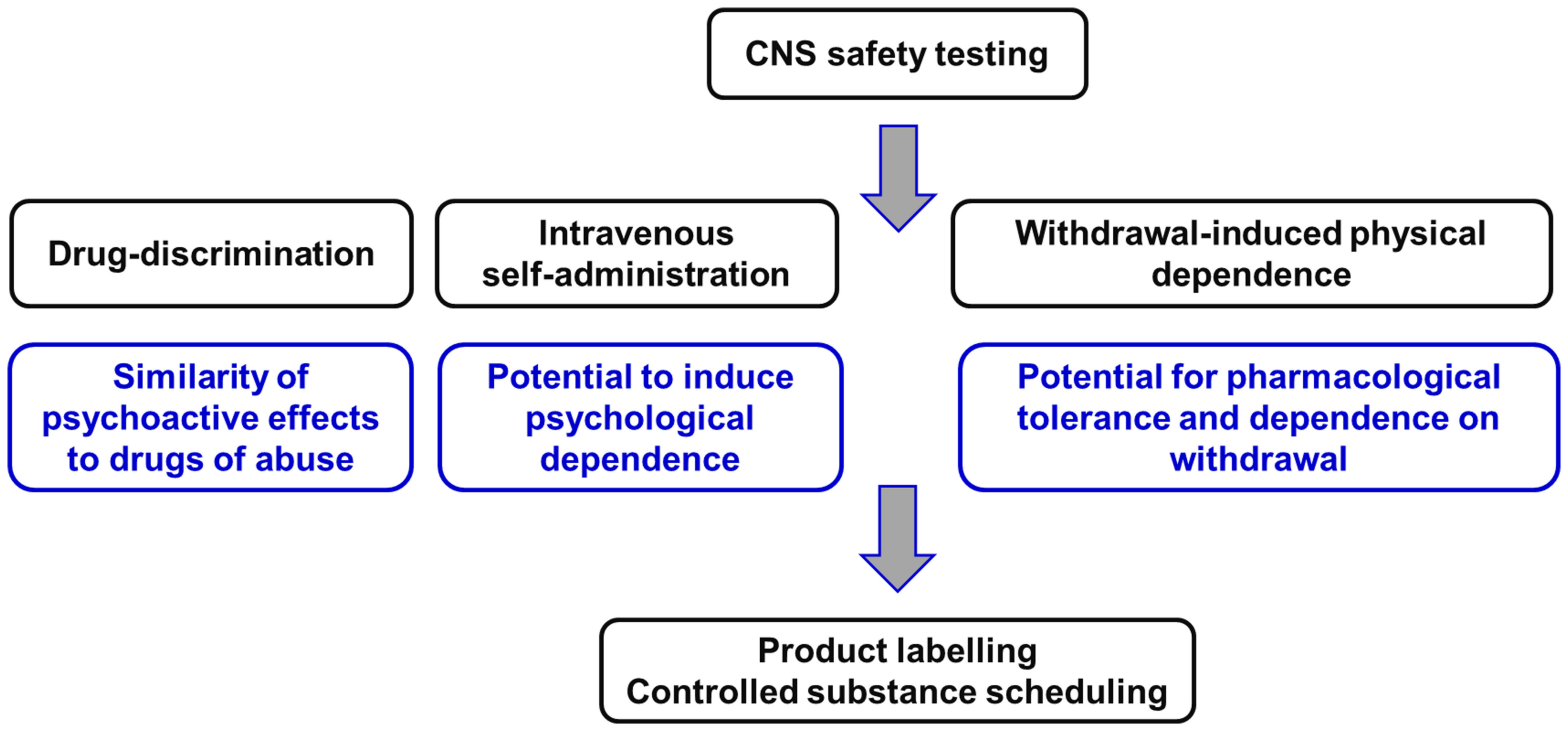
Non-clinical experiments to evaluate the potential of novel CNS-active compounds for human abuse and to cause physical dependence on withdrawal. This package of Safety Pharmacology studies is described in the guidance documents issued by the Committee for Medicinal Products for Human Use (58) of the European Medicines Agency [EMA] (58) and the Center for Drug Evaluation and Research (59) of the US Food and Drug Administration [FDA] (59). Figure created by the authors.

The two animal models used to evaluate abuse potential of CNS-active drugs are: (i) drug-discrimination and (ii) intravenous self-administration (**Figure 2**).

The drug-discrimination test determines whether the psychoactive effects produced by the drug-candidate are identical to those of those produced by a known substance of abuse (positive control) that the animals have been trained to recognize and differentiate from “placebo” (drug vehicle; negative control). Testing takes place in an operant box and relies on the responses of animals in a 2-choice paradigm that is signaled either by lever-presses or nose-pokes. Rats are the species most frequently employed, but mice or primates are used in some variants of the model. The subject is trained using food rewards to respond consistently on one of the levers after administration of the training drug, and to respond on the other lever after being administered with vehicle. Once the subject can reliably recognize the psychoactive effect of the training drug and differentiate it from vehicle, test compounds can be substituted in the model. The animal can signal via its operant responses whether the psychoactive effect produced by the test compound is similar to the training substance of abuse (>80% responding on the drug-assigned lever [generalization]) or not (<20% responding on the vehicle-assigned lever [no generalization]). Some similarity between the psychoactive effect of test compound and the training drug is shown by mixed responding on both levers (>20% but <80% responding on the drug-assigned lever [partial generalization]).

As discussed later in this section the psychoactive cue elicited by substances of abuse is pharmacologically specific. This specificity is critical when interpreting results from a drug-discrimination test. If a test compound fails to generalize to the cue produced by the abused drug used to train the animals, it implies their psychoactive effects are not similar. It does not follow that the test compound does not produce psychoactive effects that could support abuse in humans. An intrinsic assumption of the model is the discriminative cue produced by the training substance of abuse is identical to the one underpinning its abuse potential. This point will be explored when we review the published experimental findings obtained with psychoactive and non-psychoactive cannabinoids. The use of the drug-discrimination technique as a tool for assessing abuse potential of CNS-active compounds including its relative strengths and weaknesses has been reviewed by us and other researchers (61–67).

Early researchers reported that the Δ^9^-THC discriminative cue was not blocked by opiate, dopaminergic, serotonergic, adrenergic, or cholinergic receptor antagonists (68), revealing a lack of involvement of these neurotransmitter systems in its CNS effects. After the discovery of selective cannabinoid antagonists, it was shown that the psychoactive effects of Δ^9^-THC were blocked by the selective CB1 receptor agonist, rimonabant (69–71). Rimonabant also abolished the discriminative cues of various cannabinoids that generalize to THC, i.e., WIN 55,212 (70–72), HU-210 (71), AM-678 (73), AM-5983 (72) and R-methanandamide (73). Lastly, the discriminative cue produced by the CB1 receptor agonist, JWH-018, was blocked by rimonabant, but not by the selective CB2 receptor antagonist, SR-144528 (43). Together, the findings show that although Δ^9^-THC is a CB1 and CB2 partial agonist, activation of the CB1 receptor subtype is solely responsible for producing its psychoactive effect.

A wide range of naturally occurring and synthetic cannabinoids have been tested for their ability to generalize to Δ^9^-THC in the drug-discrimination model (**Table 4**). The results have been obtained from experiments performed in monkeys, rats, mice and pigeons and they show excellent consistency regardless of the species employed in the test. The other major psychoactive phytocannabinoids, i.e., Δ^8^-tetrahydrocannabinol (Δ^8^-THC) and cannabinol (CBN), substitute for Δ^9^-THC in drug-discrimination. Δ^8^-THC [ED_50_ = 2.02 mg/kg] and CBN [ED_50_ = 6.77 mg/kg] are less potent than Δ^9^-THC [ED_50_ = 0.88 mg/kg] in the model (68), indicating they are less psychoactive. The (+)-enantiomer of Δ^8^-THC, which is not the naturally occurring optical enantiomer, failed to generalize to the Δ^9^-THC cue. Consistent with the hypothesis that the CB1 receptor mediates the psychoactive effects of Δ^9^-THC, CBD did not generalize to its discriminative cue in either rats or pigeons.

**Table 4:** Profile of psychoactive and non-psychoactive cannabinoids in Δ^9^-THC-cued drug-discrimination testing.

Compound	Discriminative cue	Species	Training dose (mg/kg)	Result	ED_50_ (mg/kg)	Source
Psychoactive phytocannabinoids						
Δ^9^-THC	JWH-018	Mouse	0.3 mg/kg, i.p.	Yes	1.28	(43)
Δ^9^-THC	AM-2389	Rat	0.56 mg/kg,i.p.	Yes	0.844	(74)
Δ^9^-THC	AM-5983	Rat	0.18 mg/kg, i.p.	Yes	0.264	(72)
(–)Δ^8^-THC	Δ^9^-THC	Rat	3.0 mg/kg, i.p.	Yes	ND	(75)
(–)Δ^8^-THC	Δ^9^-THC	Rat	3.2 mg/kg, i.p.	Yes	2.02	(68)
(-)Δ^8^-THC	Δ^9^-THC	Pigeon	0.25 mg/kg, i.m.	Yes	ND	(76)
(+)Δ^8^-THC	Δ^9^-THC	Rat	3.0 mg/kg, i.p.	No	ND	(75)
CBN	Δ^9^-THC	Rat	3.2 mg/kg, i.p.	Yes	6.77	(68)
CBN	Δ^9^-THC	Rat	3.0 mg/kg, i.p.	Yes	8.39	(77)
CBN	Δ^9^-THC	Pigeon	0.25 mg/kg, i.m.	Yes	ND	(76)
CBN	Δ^9^-THC	Pigeon	0.56 mg/kg, i.p.	Yes	14.1	(77)
Non-psychoactive phytocannabinoids						
CBD	Δ^9^-THC	Rat	3.2 mg/kg, i.p.	No	ND	(68)
CBD	Δ^9^-THC	Pigeon	0.25 mg/kg, i.m.	No	ND	(76)
CBG	*No data*					
CBC	*No data*					
Psychoactive synthetic cannabinoids						
Nabilone	Δ^9^-THC	Rat	3.2 mg/kg, i.p.	Yes	0.36	(68)
(-)Δ^8^-THC-DMP	Δ^9^-THC	Rat	3.0 mg/kg, i.p.	Yes	ND	(75)
R-Methanandamide	Δ^9^-THC	Rat	0.18 mg/kg, i.p.	Yes	5.41	(73)
R-Methanandamide	Δ^9^-THC	Rat	2.0 mg/kg, i.p.	Yes	1.42	(78)
*(R)-Methanandamide*	*AM-5983*	*Rat*	*0.18 mg/kg, i.p.*	Yes	3.37	(72)
5F-EDMB-PINACA	Δ^9^-THC	Mouse	5.6 mg/kg, i.p.	Yes	0.022	(34)
5F-MDMB-PICA	Δ^9^-THC	Mouse	5.6 mg/kg, i.p.	Yes	0.17	(34)
5F-MMB-PICA	Δ^9^-THC	Mouse	5.6 mg/kg, i.p.	Yes	0.323	(34)
AB-CHMINACA	Δ^9^-THC	Mouse	5.6 mg/kg, i.p.	Yes	0.09	(79)
AM-2201	Δ^9^-THC	Mouse	3.0 mg/kg, i.p.	Yes	0.11	(80)
*AM-4971*	*AM-5983*	*Rat*	*0.3 mg/kg, i.p.*	Yes	0.041	(72)
*AM-5760*	*AM-5983*	*Rat*	*0.3 mg/kg, i.p.*	Yes	0.520	(72)
*AM-5983*	*AM-2389*	*Rat*	*0.56 mg/kg, i.p.*	Yes	0.007	(74)
AM-678	Δ^9^-THC	Rat	0.18 mg/kg, i.p.	Yes	0.044	(73)
AMB-FUBINACA	Δ^9^-THC	Mouse	5.6 mg/kg, i.p.	Yes	0.04	(79)
APP-BINACA	Δ^9^-THC	Mouse	5.6 mg/kg, i.p.	Yes	54.1	(34)
CP 47,497	Δ^9^-THC	Mouse	5.6 mg/kg, i.p.	Yes	0.25	(81)
CP 47,497-C8-homologue	Δ^9^-THC	Mouse	3.0 mg/kg, i.p.	Yes	0.83	(80)
CP 55,940	Δ^9^-THC	Monkey	0.1 mg/kg, i.v.	Yes	0.003	(71)
CP 55,940	Δ^9^-THC	Rat	2.0 mg/kg, i.p.	Yes	0.005	(78)
CP 55,940	Δ^9^-THC	Mouse	5.6 mg/kg, i.p.	Yes	0.03	(79)
FUB-144	Δ^9^-THC	Mouse	5.6 mg/kg, i.p.	Yes	3.01	(34)
FUB-AKB48	Δ^9^-THC	Mouse	5.6 mg/kg, i.p.	Yes	1.69	(34)
HU-210	Δ^9^-THC	Monkey	0.1 mg/kg, i.v.	Yes	0.005	(71)
JWH-018	Δ^9^-THC	Rat	0.3 mg/kg, i.p.	Yes	0.23	(46)
*JWH-073*	*JWH-018*	*Mouse*	*0.3 mg/kg, i.p.*	Yes	0.69	(43)
JWH-073	Δ^9^-THC	Rat	0.3 mg/kg, i.p.	Yes	1.31	(46)
JWH-200	Δ^9^-THC	Mouse	3.0 mg/kg, i.p.	Yes	1.16	(80)
JWH-203	Δ^9^-THC	Mouse	3.0 mg/kg, i.p.	Yes	1.50	(80)
JWH-204	Δ^9^-THC	Mouse	10 mg/kg, s.c.	Partial	3.77	(69)
JWH-205	Δ^9^-THC	Mouse	10 mg/kg, s.c.	Yes	29.4	(69)
JWH-210	Δ^9^-THC	Rat	0.3 mg/kg, i.p.	Yes	1.23	(46)
JWH-250	Δ^9^-THC	Mouse	3.0 mg/kg, i.p.	Yes	1.00	(80)
MDMB-4en-PINACA	Δ^9^-THC	Mouse	5.6 mg/kg, i.p.	Yes	0.025	(34)
MMB-4en-PICA	Δ^9^-THC	Mouse	5.6 mg/kg, i.p.	Yes	1.44	(34)
MMB-FUBICA	Δ^9^-THC	Mouse	5.6 mg/kg, i.p.	Yes	0.999	(34)
SP-106	Δ^9^-THC	Rat	3.2 mg/kg, i.p.	Yes	1.06	(68)
WIN 55,212	Δ^9^-THC	Monkey	0.1 mg/kg, i.v.	Yes	0.051	(71)
*WIN 55,212*	*AM5983*	*Rat*	*0.18 mg/kg, i.p.*	Yes	0.208	(72)
Non-psychoactive synthetic cannabinoids						
JWH-320	Δ^9^-THC	Rat	3.0 mg/kg, i.p.	No	No substitution	(46)
KLS-13019	*No data*					
8,9-Dihydrocannibidiol	*No data*					

CBC, cannabichromene; CBD, cannabidiol; CBG, cannabigerol; CBN, cannabinol; Δ^8^-THC, (-)Δ^8^-tetrahydrocannabinol; Δ^9^-THC, (-)Δ^9^-tetrahydrocannabinol.

Results shown in italics show indicate experiments that did not employ Δ^9^-THC as the training drug.

ND, not determined.

Yellow shading indicates replicated data for individual compounds.

A substantial number of synthetic cannabinoids have been created. Many of these compounds not only have much higher CB1 receptor affinity than Δ^9^-THC they are also CB1 receptor full agonists (**Tables 2**, **3**). As shown in **Table 4**, these synthetic cannabinoids without exception generalize to the Δ^9^-THC cue and with far greater potency than Δ^9^-THC. Some of these synthetic cannabinoids with powerful CB1 receptor agonist properties, e.g., JWH-018, JWH-073, JWH-200, JWH-250, CP-47,497, and AM-2201, have been illicitly sold as quasi-legal alternatives to cannabis under the names “K2” or “Spice”. Following legislation to outlaw the production, distribution, and possession of these compounds in many countries, a “second generation” of synthetic cannabinoids has emerged with chemical structures that are sufficiently different from the Δ^9^-THC to ensure they elude the legal controls governing Δ^9^-THC and other psychoactive phytocannabinoids. This strategy permits each molecule to remain “quasi-legal” up to the point when it is declared to be an illegal controlled substance, Some of these highly potent synthetic cannabinoids, e.g. 5F-EDMB-PINACA, FUB-AKB48, FUB-144, MDMB-4en-PINACA, have been linked to human fatalities or other serious harms; all of these novel synthetic cannabinoids generalize to Δ^9^-THC (**Table 4**), but the potency increase of some of these compounds relative to Δ^9^-THC is staggering. Based on ED_50_ values in the drug-discrimination test, 5F-MDMB-PICA, 5F-EDMB-PINACA and MDMB-4en-PINACA are at least 100-fold more potent than Δ^9^-THC (34).

The Δ^9^-THC analogue, nabilone and CBD (Epidiolex™) are both approved as prescription medicines. Neither drug has undergone safety pharmacology assessment in Δ^9^-THC-cued drug-discrimination testing. Nabilone [ED_50_ = 0.36 mg/kg] is approximately twice as potent as Δ^9^-THC [ED_50_ = 0.88 mg/kg] (68). Since CBD (Epidiolex™) was mildly sedative in animals and humans at high doses, its ability to generalize to midazolam was determined, but the compound was negative in this test procedure (82).

The combined findings show that Δ^9^-THC, which is the major psychoactive cannabinoid in cannabis, is active and potent [ED_50_ < 1.0 mg/kg] in the CB1 receptor agonist-cued drug discrimination test. Δ^8^-THC and CBN mimic the psychoactive effects of the Δ^9^-THC cue, but are 2-5-fold less potent. Given they are also less abundant in the cannabis plant than Δ^9^-THC and have lower potency, their contribution to the psychoactive properties of cannabis is relatively limited. Consistent with the view that CBD does not produce Δ^9^-THC-like psychoactive effects, CBD did not generalize to Δ^9^-THC in drug-discrimination. The intoxicating and harmful effects of cannabis are, however, greatly exceeded by those of the first and second generation of synthetic CB1 agonists present in illegal substances like K2 and Spice.

The intravenous self-administration model determines whether the psychoactive effects produced by the test compound are rewarding, leading to psychological dependence that will initiate craving or drug-seeking. It is a motivational task in which the animal is required to work (lever-press or nose-poke) to receive a reward (a drug infusion). If the experience of taking the drug is rewarding, the subject will be motivated to work to receive further drug infusions (positive reinforcement). On the other hand, if the experience is non-rewarding (non-reinforcing) or dysphoric (negatively reinforcing), the subject will not lever-press to self-administer drug infusions. This test is also conducted in 2-lever operant boxes. One lever is programmed to deliver small unit-doses of the positive reinforcing substance of abuse that is used to train the animals. The other lever is usually available, but pressing it produces no programmed consequences; it is there to investigate non-specific effects on lever selection and motor function. The abused substance used to train the rats is usually a strong positive reinforcer, e.g., heroin or cocaine. Once the animal reproducibly self-administers consistent levels of the training drug, saline (“placebo” [non-reinforcer]) is substituted for the training drug in the experimental sessions until operant responding has been extinguished (extinction). This process produces an experimental subject that is motivated to work to earn infusions of the rewarding drug but not vehicle. At this point, the test compound can be introduced into the model to determine whether it will maintain levels of self-administration significantly greater than vehicle (non-reinforcer). If it does, the result indicates the test compound produces a positive reinforcing experience that could support abuse in humans and ultimately lead to psychological dependence.

It is important to appreciate that the substance of abuse used to train the animals maintains self-administration because its effects are rewarding, and not because it is either a stimulant or a sedative. The follow-on is if a test compound has positive reinforcing properties, it will substitute for the training drug irrespective of its pharmacological mechanism of action and behavioral profile. This point is amply demonstrated by the observation that a group of rats trained to self-administer the opioid euphoriant, heroin, will readily self-administer MDMA, (-)-pentazocine, butorphanol, benzodiazepines, methohexital, or cocaine when substituted for heroin (65, 82–84); data on file.

In its simplest form, this operant task is performed using a fixed ratio (FR) reward schedule, i.e., the subject has to press the active lever a fixed number of times to receive each drug infusion. When a test compound maintains self-administration at levels significantly greater than vehicle, the result indicates it is a positive reinforcer. What the result does not reveal is how reinforcing the test compound is relative to established recreationally abused drugs. As an “effort versus reward” task, intravenous self-administration can be adapted to determine the magnitude of the positive reinforcing effect of the test article relative to established substances of abuse. This is achieved by incrementally increasing the number of operant responses that the animal makes to earn each additional drug infusion in the test session (progressive ratio [PR] reinforcement schedule). At the point where the value of the reward no longer justifies the effort (lever-presses) to earn it (break-point), the animal will cease to respond to self-administer more infusions. By applying PR/break-point analyses in addition to FR testing in intravenous self-administration experiments, important additional information about the relative reinforcing efficacy of the test compound can be gathered. In rats, compounds that are strong reinforcers support PR/break-points = 70-100 lever- presses/infusion, moderate reinforcers = 30-40 lever-presses/infusion, weak reinforcers = 20-30 lever-presses/infusion, and vehicle = ~10 lever-presses/infusion (65).

We would also draw the reader’s attention to other reviews on the general methodological aspects of intravenous self-administration testing including refinements to improve the sensitivity and predictive validity of the model (63–66, 85–87).

In general, there is excellent agreement between the ability of drugs to serve as positive reinforcers in animal self-administration tests and their potential for abuse in humans. It is unfortunate that Δ^9^-THC was for many years considered to be an exception (the other being the 5-HT_2A_ agonist psychedelics). As reviewed by Tanda (2016) (88), many attempts were made in the 1970s and 1980s to show that the reinforcing effect of Δ^9^-THC in humans back-translated by maintaining Δ^9^-THC self-administration in animals. Experiments were conducted in rhesus monkeys and rats using either treatment-naïve animals to determine whether they would acquire Δ^9^-THC self-administration, or in drug-experienced animals to see if Δ^9^-THC would substitute for a reinforcing drugs like heroin or cocaine (88). None of these established approaches was successful (88). Many reasons for this failure have been put forward without achieving consensus on the matter. In our view, the physicochemical characteristics of Δ^9^-THC make it particularly unsuitable for use in self-administration experiments. The model requires relatively high concentrations of test compound in aqueous solution and because Δ^9^-THC is very hydrophobic, it makes sufficient quantities of the unadulterated Δ^9^-THC available to the animals a major challenge.

Greater success in Δ^9^-THC self-administration experiments has been achieved using squirrel monkeys which are a New World species of primate. Using cocaine-trained squirrel monkeys Tanda and colleagues (89) demonstrated that these animals would consistently self-administer Δ^9^-THC on a relatively undemanding FR10 schedule of reinforcement. Rimonabant decreased Δ^9^-THC self-administration which is consistent with the discriminative cue and the reinforcing effect of Δ^9^-THC being mediated via CB1 receptor agonism. Cocaine training is not essential for squirrel monkeys to self-administer Δ^9^-THC because this phenomenon also occurs in drug-naïve animals (90); this is an important finding because a compound needs to possess reasonably strong reinforcing properties to initiate and maintain self-administration. There is also evidence to support the view that the observed lack of reinforcing effect of Δ^9^-THC in other species was because its physicochemical properties make it unsuitable for self-administration by the intravenous route. Freels et al. (2020) (91) investigated the reinforcing effect of a Δ^9^-THC-rich cannabis extract [28.4% THC, 1.8% CBN, and 1.38% CBD] and observed that when it was presented in “vaped” form, this extract initiated and maintained self-administration in rats on a reasonably demanding FR4 schedule of reinforcement. The PR/break-point for the reinforcing effect of the Δ^9^-THC-rich cannabis extract was ~20 compared with ~9 nose-pokes for saline, indicating moderate/weak reinforcing effects. These recent data support the view that Δ^9^-THC is a moderate level positive reinforcer in rodents and primates which is consistent with its abuse profile in humans.

**Figure 3:**
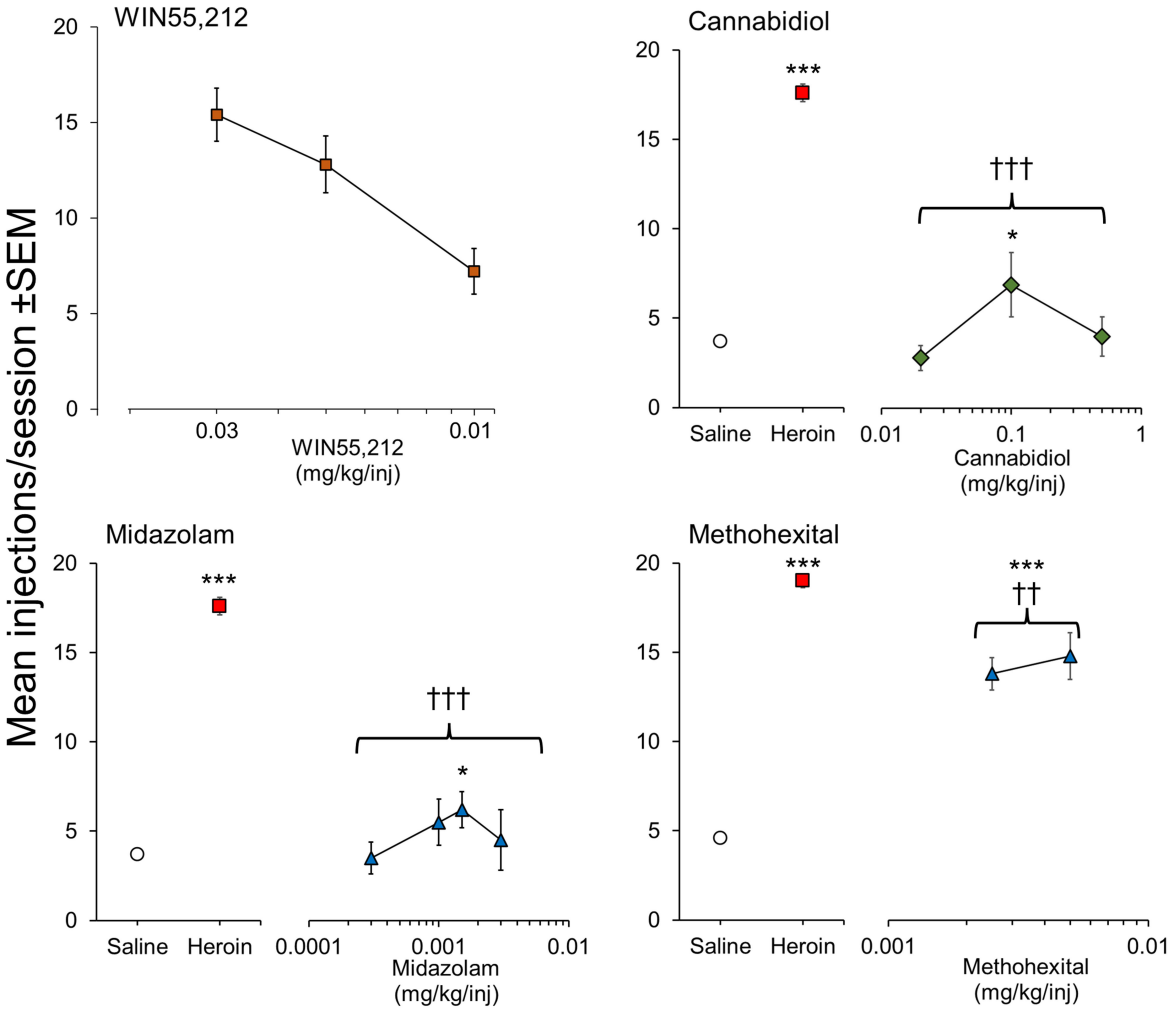
Positive reinforcing effects of WIN 55,212 (synthetic psychoactive cannabinoid) and CBD (non-psychoactive cannabinoid) evaluated by intravenous self-administration testing in male, Sprague-Dawley rats. Drug doses are shown on the horizontal axis with the mean number of infusions taken by the rats when self-administration responses were stable on the vertical axis. For CBD, midazolam and methohexital, rats were initially trained to self-administer heroin (15 ug/kg/injection) on a FR3 reinforcement schedule followed by extinction of responding on saline. WIN 55,212 was tested in drug-naïve rats on a FR3 reinforcement schedule. CBD: n = 8/dose group. WIN 55,212: n = 8/dose group. Midazolam: n = 7-17/dose group. Methohexital: n = 7/dose group. Data on file. Figure created by the authors. Significantly different from saline * p<0.05, *** p<0.001. Significantly different from heroin ††† p < 0.001.

WIN 55,212 is a potent CB1/CB2 agonist that initiated and maintained self-administration in rats on a FR1 schedule (92). More recently, Lefever et al. (2014) (93) showed that WIN 55,212 would maintain self-administration in rats on a more demanding FR3 schedule and showed its rewarding effect was blocked by the CB1 antagonist, rimonabant. We have also conducted intravenous self-administration experiments in rats using this potent agonist and observed that it initiated and maintained self-administration on a FR3 schedule in drug-naïve rats (**Figure 3**), PR/break-point determination revealed that WIN 55,212 had moderate/weak reinforcing properties (PR/break-point = 22.5 ± 4 lever-presses/infusion [n = 6] versus Saline =10.4 ± 0.8 lever-presses/infusion [n = 31]). On that basis, we predict that WIN 55,212 has greater reinforcing efficacy than Δ^9^-THC (91); a hypothesis supported by the finding that when Δ^9^-THC was substituted in WIN 55,212-trained rats, it still failed to serve as a reinforcer (93). JWH-018, another potent, synthetic CB1/CB2 agonist, was reliably self-administered by rats on a FR3 schedule; and its reinforcing effect was also blocked by rimonabant (94). These researchers replicated the positive reinforcing effect of JWH-018 in mice and confirmed this effect was exclusively mediated by CB1 receptor activation (95). In contrast, Tampus et al. (2015) (96) reported that 3 related cannabinoids, i.e., JWH-030, JWH-175, and JWH-176, failed to support self-administration in rats on a FR1 schedule. On reviewing their methodology, we noted that the rats were not mildly food restricted which is an important requisite for initiating robust self-administration of drugs. Viewed overall, it can be concluded that Δ^9^-THC serves as a moderate reinforcer in animals, but the potent, synthetic CB1 agonists are far more reinforcing than the psychoactive cannabinoids present in cannabis, and consequently, pose a greater risk for the development of psychological dependence.

As part of the abuse potential evaluation of Epidiolex™ (CBD), we investigated its ability to serve as a positive reinforcer in a group of rats trained to self-administer heroin on a FR3 schedule and observed a very small reinforcing signal (**Figure 3**). The magnitude of the abuse signal was minimal in comparison to heroin (positive control) and similar or less than the reference comparators, diazepam and methohexital (**Figure 3**). In a follow-up experiment in 5 midazolam-trained rhesus monkeys, Epidiolex™ (CBD) was found to be non-reinforcing (82, 91) investigated a CBD-enriched extract (59.34% CBD, 2.1% cannabichromene [CBC], 1.1% cannabigerol [CBG], 1.16% Δ^9^-THC, and <0.01% tetrahydrocannabivarin [THCV] and CBN) and similarly observed a trend towards increased self-administration of this overwhelmingly non-psychoactive mixture of phytocannabinoids. A number of synthetic CBD analogues are now emerging, e.g., KLS-13019, but none of them has been evaluated in self-administration.

The standard safety pharmacology program of abuse potential tests supplemented by pharmacological characterization has identified the CB1 receptor as the mediator of the psychoactive and reinforcing effects of plant-derived and synthetic cannabinoids. These non-clinical findings predict that Δ^9^-THC and other psychoactive cannabinoids in cannabis are positive reinforcers with moderate potential to induce psychological dependence. These results also support the view that phytocannabinoids like CBD, CBC and CBG pose no abuse risks to humans. The content of Δ^9^-THC in cannabis has substantially increased over time and, therefore, so has its potential for abuse and harm. However, Δ^9^-THC and the other psychoactive cannabinoids present in cannabis are CBI partial agonists and pose far less of a risk than the potent CB1 full agonists that are being synthesized and illegally sold.

The standard experimental procedure that is used to determine whether a CNS-active drug induces a syndrome of dependence on withdrawal is to administer it for a period of at least 21-28 days to allow neuro-adaptation to occur, abruptly terminate dosing, and monitor for signs of withdrawal over the following 7 days (or at least 5 biological drug half-lives). Experiments are generally conducted in rats using a 4-cohort, parallel design with 2 doses of the test compound (the first in the therapeutic or pharmacological range and the second 2-3 times higher), a positive control (an opiate or benzodiazepine) and a negative control (vehicle). Withdrawal signs span a broad spectrum of signs comprising physiological (e.g., food and water intake, body weight and body temperature), behavioral (e.g., teeth-chattering, writhing, tremors, posture, gait, vocalization), and physical (e.g., loss of condition, piloerection, stained fur and nose) events. Although the focus is physical dependence, many of these signs would also indicate psychological distress or stress. The alternative approach to monitor withdrawal is to administer the test compound repeatedly for several days and then “precipitate” withdrawal by administering an antagonist. The spontaneous withdrawal model is preferred over precipitated withdrawal because it is more clinically relevant. In the case of the cannabinoids, there is an additional confounder to using precipitated withdrawal testing. When this type of experiment is conducted using an opiate as the positive control and naloxone as the precipitated withdrawal drug, the latter induces no effects when administered alone. However, a CB1 antagonist induces many of the withdrawal signs produced by spontaneous withdrawal after repeated CB1 agonist administration (97, 98), making identification and interpretation of the findings extremely difficult. The other confounder in many of these studies is high cannabinoid doses have been used as a substitute for an adequate duration of dosing (52, 97), which also impairs translational validity of the results. Given those limitations, Paronis et al. (2022) (99) investigated spontaneous withdrawal in groups of mice that were treated for 5 days with Δ^9^-THC (20-36 mg/kg/day), the synthetic psychoactive CB1/CB2 agonist, AM-2389 (0.06-0.1 mg/kg/day), or saline. The results showed that pharmacological tolerance occurred rapidly on repeated daily administration of Δ^9^-THC or AM-2389. However, there was no rebound increase in body temperature and no change in locomotor activity on withdrawal. The only behavioral sign of withdrawal was increased paw tremors, and this effect was more pronounced in the mice receiving AM-2389 than Δ^9^-THC. This would be classified as a very mild withdrawal syndrome compared to one caused by the benzodiazepines/barbiturates or opiates. In an earlier study, Aceto et al. (1996) (97) observed mild spontaneous withdrawal signs in mice after 4 days of treatment with very high doses of Δ^9^-THC (12.5 mg/kg escalating to 100 mg/kg), but not pharmacologically relevant doses (0.5 mg/kg escalating to 4.0 mg/kg). Oliva et al. (2003) (100) investigated the spontaneous withdrawal effects in mice after 7-days of administration of CP-55,940 (0.5 mg/kg b.i.d.). There was rapid tolerance to the pharmacological effects of CP-55,940 in the dosing phase, but only a mild syndrome in withdrawal. It lasted approximately 3 days and consisted of moderate changes in locomotor activity, rearing, grooming wet-dog shakes, body rubbing and digging. Increased plasma corticosterone levels indicated the syndrome was stressful to the mice. All of these studies have consistently shown rapid tolerance to CB1 agonists, which in turn resulted in a mild/moderate withdrawal syndrome. Even the more pronounced withdrawal signs seen with precipitated withdrawal (97, 98) are mild in comparison to spontaneous opiate or benzodiazepine withdrawal (82, 101, 102).

Withdrawal after discontinuation of Epidiolex™ (CBD) was determined in groups of male and female rats and in juveniles as well as adults (82). Epidiolex™ (CBD) (40 or 200 mg/kg/day) was compared against morphine (64 mg/kg b.i.d.), diazepam (40 mg/kg b.i.d.), or vehicle administered for 19 days with withdrawal signs monitored for 8 days after discontinuation of dosing. No physiological or behavioral signs of withdrawal were seen after discontinuation of Epidiolex™ (CBD). In contrast, changes in body temperature and body weight, food intake, and adverse clinical signs were observed in the groups discontinued from morphine or diazepam. We could find no other published reports of spontaneous withdrawal studies having been conducted on other non-psychoactive synthetic or phytocannabinoids.

Together, the findings show that although pharmacological tolerance develops rapidly to the actions of the psychoactive cannabinoids, it results in a mild/moderate withdrawal syndrome. Consistent with other aspects of cannabinoid pharmacology, the effects of Δ^9^-THC and other psychoactive phytocannabinoids are less pronounced than the potent synthetic CB1/CB2 full agonists. The evidence for CBD shows no potential for withdrawal-induced dependence. Although there is no experimental evidence, it can also be reasonably predicted that other non-psychoactive cannabinoids pose no withdrawal risks.

## Studies in drug-experienced human volunteers

The human abuse potential of Epidiolex™ (CBD) (2) was evaluated in a single-dose, randomized, placebo- and active-controlled, crossover trial in a group of healthy recreational polydrug users. In this trial, 750 mg (clinical dose b.i.d.), 1500 mg (2x clinical dose) and 4500 mg (6x clinical dose) CBD were compared against synthetic Δ^9^-THC (dronabinol; 10 mg and 30 mg), alprazolam (2 mg), and placebo (103). The highest recorded maximum “drug liking at this moment” (primary endpoint) score occurred in the 30 mg dronabinol session, but statistically significant increases were also evoked by both doses of dronabinol or alprazolam. The scores for all doses of CBD remained in the neutral (placebo) zone. Statistically significant abuse signals produced by dronabinol and alprazolam were also present on the secondary endpoints of “take drug again” and “overall drug liking”. CBD did not separate from placebo on the former but produced small increases at 2 doses on the latter. The effects of CBD were not significantly different from placebo on a range of other measures including the alertness/drowsiness scale. In contrast to dronabinol and alprazolam, CBD did not cause cognitive impairment. This rigorously controlled trial in a population of recreational drug users not only differentiates CBD from Δ^9^-THC in abuse potential terms, it also provides evidence to demonstrate that CBD is not psychoactive. The decision not to classify Epidiolex™ (CBD) as a controlled drug was largely based on evidence from this clinical trial.

## Summary

To put the abuse and dependence results for the naturally occurring and synthetic cannabinoids into a broader context, in **Table 5**, we have compared them against various other pharmacological classes of substance of abuse.

**Table 5:** Relative abuse/dependence risks of naturally occurring and synthetic cannabinoids.

Drug class	Liability			Overall risk assessment
	Psychological dependence	Pharmacological tolerance	Physical dependence on withdrawal	
Δ^9^-THC	##	###	##	Moderate
Other naturally occurring CB1/CB2 agonists	#	###	#/0	Low
Synthetic CB1/CB2 agonists	###	###	##	Moderate
CBD	0	0	0	0
Other non-psychoactive cannabinoids	0	0	0	0
Nicotine	####	###	###	High
Opiates	####	###	####	High
Stimulants (e.g. cocaine, methamphetamine)	####	##	#/0	High
Alcohol	##	##	###	High/Moderate
Barbiturates	##	###	###	Moderate
Benzodiazepines	##/#	###	###	Moderate
Entactogens (e.g. MDMA)	##	#	#/0	Moderate/low
Psychedelics (e.g. LSD, psilocybin)	#/0	####	0	Low

Risk: #### = very high; ### = high; ## = moderate; # = low and 0 = no risk.

ND, no data.

Yellow shading indicates replicated data for individual compounds.

## Pharmacological interaction between Δ^9^-THC and CBD

Δ^9^-THC and CBD are the two major pharmacologically active compounds present in cannabis. The interplay between these two phytocannabinoids is of particular interest because there has been much speculation about CBD modulating some of the psychoactive effects of Δ^9^-THC and also about reduction of its potential for harm. We have explored this topic, and the evidence contained the nonclinical and clinical literature is discussed in this section.

## Nonclinical evidence

### Pharmacology

Moore & Weerts (2022) (104) found that the tetrad effects of Δ^9^-THC on antinociception (tail flick and von Frey tests), hypothermia, hyper- and hypolocomotion, and catalepsy were comparable in male and female rats and persisted for more than 7 hours. In contrast, CBD, which is devoid of CB1 agonist activity, did not evoke the classic cannabinoid tetrad and moderately increased pain sensitivity and evoked sex-dependent effects on body temperature and locomotor activity. Taffe et al. (2015) (105) used radiotelemetry to investigate whether high or low content CBD cannabis strains modulated hypothermia or hypolocomotion induced by Δ^9^-THC. When rats were treated with Δ^9^-THC alone, or with CBD in a 1:1 ratio or 3:1 ratio with Δ^9^-THC, CBD failed to reverse the hypothermia or locomotor suppression produced by Δ^9^-THC, and in the 1:1 ratio, significantly increased hypothermia.

The effects of repeated administration of Δ^9^-THC and CBD on the cognitive performance of ageing mice was studied by Sadaka et al, (2023) (106). Animals were exposed to vaporized cannabis containing ~10% Δ^9^-THC + low CBD or ~10% CBD + low Δ^9^-THC for 28 days to achieve blood levels similar to those reported in human users. Δ^9^-THC/low CBD, but not CBD/low Δ^9^-THC, impaired cognitive performance when vaped acutely, but tolerance to this effect occurred on repeated dosing. No adverse cognitive effects were observed on withdrawal from either intervention. The ability of CBD to mitigate the cognitive deficits and withdrawal signs produced by the synthetic psychoactive cannabinoid, WIN-55,212, were investigated in a conditional discrimination task, the Barnes maze, and elevated plus maze. Δ^9^-THC and WIN-55,212 produced moderate learning and memory impairments CBD (98). CBD did not affect cognitive performance and it failed to attenuate the adverse effects produced by administration of Δ^9^-THC or WIN-55,212 (98).

Cannabis exposure during adolescence may lead to neurobiological changes that affect brain functions and increase the risk of cannabis use disorder during adulthood. Adult female rats who had been exposed to long-term administration of pure Δ^9^-THC during adolescence showed short-term memory deficits in the novel object recognition test, increased immobility in the forced swim test, anhedonia and reduced social interaction behavior (107). These effects on anxiety-like behaviors in the social interaction test were prevented by administering a Δ^9^-THC/low CBD (3:1 Δ^9^-THC: CBD) formulation. In contrast, repeated treatment with a CBD/low Δ^9^-THC (33:1 CBD: Δ^9^-THC) formulation during adolescence did not induce depressive-like behaviors in the forced swim test, social behavior deficits nor anxiety-like behaviors in adults but did produce short-term memory deficits and anhedonia in the long-term. Exposure in adolescence to pure Δ^9^-THC down-regulated CB1 receptors in the prefrontal cortex of the adults. This CB1 receptor decrement was prevented when the Δ^9^-THC/low CBD combination was administered. Together the findings suggest that CBD was without adverse effects and it could mitigate some of the long-term behavioral alterations induced by adolescent Δ^9^-THC exposure as well as CB1 receptor desensitization.

The interaction between CBD and Δ^9^-THC on longer-term exposure was investigated in adolescent squirrel monkeys that were treated over 4 months with high Δ^9^-THC (1 mg/kg) or high THC/high CBD (1 mg/kg + 3 mg/kg) (108). Δ^9^-THC impaired cognitive performance (repeated acquisition), but not performance in a cognitive flexibility task (discrimination reversal) (108). As expected, Δ^9^-THC also reduced motor activity and increased sedentary behavior with tolerance developing after a few weeks of daily treatment. None of the cognitive or behavioral effects of Δ^9^-THC were influenced by co-administration of CBD (108).

### Drug-discrimination

CBD does not generalize to the discriminative cue produced Δ^9^-THC in rats (68) or pigeons (76). Interestingly, Hiltunen & Jarbe (1986) (109) found that the effect of CBN to generalize to a Δ^9^-THC cue was reduced when it was administered with CBD in a time- and dose-dependent manner. Vann et al. (2008) (110) assessed CBD and Δ^9^-THC alone and in combination in drug-discrimination in rats and in conditioned place preference/aversion in mice. CBD did not have effects in either procedure. Although none of the CBD/Δ^9^-THC dose ratios (0.3-30 mg/kg CBD; 0.3-10 mg/kg Δ^9^-THC) altered the discriminative stimulus effects of Δ^9^-THC, CBD/Δ^9^-THC at dose ratios of 1:1 and 1:10 reversed the conditioned place aversion produced by a single dose of Δ^9^-THC suggesting that CBD can attenuate the aversive effects of Δ^9^-THC.

### Intravenous self-administration

There are concerns that exposure to cannabis in adolescence could increase the risk of developing dependence on the drug later in life. To address concerns that exposure to cannabis in adolescence may increase the risk of developing dependence on the drug later in life, Scherma et al. (2016) (111) investigated whether exposure to Δ^9^-THC in adolescent rats could enhance the reinforcing effects of cannabinoids in adults. Rats were treated with increasing doses of Δ^9^-THC for 11 days (post-natal days 45–55) before being trained to intravenously self-administer WIN55,212 as adults. In the Δ^9^-THC-exposed rats, the acquisition of WIN55,212 self-administration was enhanced. In addition, there was a decreased effect of WIN55,212 to stimulate firing of dopamine neurons in the ventral tegmental area or to increase extracellular dopamine in the nucleus accumbens shell, indicating a blunting of the rewarding effect of CB1 agonists. Whether CBD can alleviate these effects is not known, but CBD co-formulated with Δ^9^-THC in 1:1 and 1:10 dose ratios did not influence Δ^9^-THC self-administration by male and female rats (112).

### Withdrawal-induced physical dependence

The possibility CBD can ameliorate the cannabis withdrawal syndrome was investigated by Myers et al, (2019) (98). Repeated administration of CBD alone to mice with precipitated withdrawal induced by rimonabant elicited no signs of dependence. The dependence signs produced by antagonist precipitated withdrawal after repeated administration of Δ^9^-THC or WIN-55,212 were not attenuated by chronic administration of CBD.

### Nonclinical summary

Overall, the findings from the non-clinical studies provide scant evidence to support the hypothesis that CBD can influence the abuse or dependence potential of cannabis or synthetic CB1 receptor agonists or attenuate their adverse effects on psychomotor and cognitive function.

## Clinical studies

### Clinical pharmacology

The pharmacokinetics and pharmacodynamics of orally administered high Δ^9^-THC/zero CBD (20mg Δ^9^-THC with no CBD), or high Δ^9^-THC/high CBD (20mg Δ^9^-THC + 640mg CBD) cannabis extracts were investigated in a randomized, placebo-controlled, double-blind, crossover study in 18 adults with experience of cannabis use (113). High Δ^9^-THC/high CBD produced increased C_max_ and AUC values for Δ^9^-THC and its metabolites (11OH-Δ^9^-THC, and Δ^9^-THC-COOH) relative to high Δ^9^-THC/zero CBD. The high Δ^9^-THC/high CBD combination also increased self-reported anxiety, sedation and memory difficulty, increased heart rate, and produced a more pronounced impairment of cognitive and psychomotor performance compared with high Δ^9^-THC/zero CBD or placebo. Stronger adverse effects were elicited from a CBD-dominant cannabis extract compared Δ^9^-THC alone which contradicts claims that CBD can reduce adverse effects of Δ^9^-THC. Based on the pharmacokinetic results, inhibition of Δ^9^-THC metabolism by CBD could be the responsible mechanism.

### Cognitive tests

In a double-blind, randomized, crossover trial in 48 light or heavy users of cannabis, subjects inhaled Δ^9^-THC (8mg), CBD (16mg) or Δ^9^-THC (8mg) + CBD (16mg) (Δ^9^-THC/CBD). Δ^9^-THC significantly increased scores on the Psychotomimetic States Inventory [PSI] (perceptual distortions, cognitive disorganization [psychotic adverse events]), increased negative thoughts on the Brief Psychiatric Rating Scale and impaired working memory (114). CBD alone reduced PSI scores but only in light cannabis users. When co-administered with Δ^9^-THC, CBD did not influence its adverse effects on the CNS. In a separate study to assess whether pre-treatment with CBD attenuated Δ^9^-THC-induced psychotic effects and cognitive impairment, 22 healthy subjects with experience of cannabis use received oral CBD (600mg) and 26 matched subjects received placebo intravenous injection of Δ^9^-THC (1.5 mg) (115). CBD did not influence any Δ^9^-THC-induced changes on the Positive and Negative Syndrome Scale (PANSS), but clinically significant positive psychotic symptoms were fewer in the CBD group compared with placebo. Post-Δ^9^-THC paranoia rated on the State Social Paranoia Scale was reduced by CBD, and in addition, episodic memory relative to baseline was poorer in the placebo-treated subjects. In a later study from this research group, 46 healthy, infrequent cannabis users were tested in a double-blind, within-subject, randomized trial of cannabis with varying CBD content (116). Subjects inhaled cannabis containing 10mg Δ^9^-THC with 0 mg CBD (0:1 CBD: Δ^9^-THC), 10 mg CBD (1:1), 20mg CBD (2:1), or 30 mg CBD (3:1). The primary outcome measure was change in delayed verbal recall on the Hopkins Verbal Learning Task, with secondary outcomes including additional cognitive tests together with subjective, pleasurable, pharmacological, and physiological effects. Δ^9^-THC without CBD (0:1) impaired delayed verbal recall, and this impairment was not reduced by administering it with any dose of CBD. Furthermore, there was no evidence of CBD influenced the effects of Δ^9^-THC on the other evaluated parameters.

Ilan et al. (2005) (117) conducted a double-blind, placebo-controlled, mixed between- and within-subject trial to investigate the contribution of different cannabinoids to the subjective, behavioral, and neurophysiological effects of inhaled cannabis. A group of 23 cannabis users were administered low Δ^9^-THC (1.8%), high Δ^9^-THC (3.6%), or placebo joints, or Δ^9^-THC joints with low or high levels of CBC (0.1% or 0.5%), or CBD (0.2% or 1.0%). Compared with the placebo joint, the Δ^9^-THC-containing cigarette produced adverse effects on mood, behavior and brain activity monitoring attentional processes during tests of working and episodic memory. The addition of CBC or CBD did not change any of the outcome measures.

The acute effects of Δ^9^-THC and CBD alone and in combination on rationale mismatch negativity (a candidate endophenotype for cognitive deficits in schizophrenia) were studied because of increasing evidence it is adversely affected by prolonged cannabis use. In a randomized, placebo-controlled, double-blind, crossover study (118), 18 frequent and 18 moderate cannabis users were administered Δ^9^-THC 8 mg, CBD 400 mg, Δ^9^-THC 8 mg + CBD 4 mg (Δ^9^-THC/low CBD), or Δ^9^-THC 12 mg + CBD 400 mg (Δ^9^-THC/high CBD) by vaporization. Δ^9^-THC or CBD given alone increased the duration and intensity amplitude of mismatch negativity in less-frequent users compared with placebo, and Δ^9^-THC also increased frequency amplitude in this group. Δ^9^-THC/low CBD attenuated the effect of Δ^9^-THC on the duration and intensity amplitude in less frequent users, while Δ^9^-THC/high CBD decreased these parameters in moderate users.

Sensory gating is the process whereby the brain reduces an evoked response to repeated stimuli. Sensory gating takes several forms including modulation of sensation and perception due to changes in arousal, recent stimulus exposure and selective attention. A study by Skosnik et al. (2018) (119) investigated whether cannabinoid administration would disrupt sensory gating using electroencephalography in humans and evoked neural oscillations using local field potentials in rats. In the randomized, placebo-controlled, double-blind part of the study, 15 experienced cannabis users received intravenous Δ^9^-THC (2.5 mg), CBD (5 mg/kg), Δ^9^-THC (2.5 mg) + CBD (5 mg) [Δ^9^-THC/CBD]. Compared with placebo, Δ^9^-THC and Δ^9^-THC/CBD disrupted sensory gating to the same extent, whereas CBD alone had no effect. The involvement of CB1 receptors was investigated in rats that were administered CP-55940 (CB1/CB2 agonist) or CP-55940 + AM-251 (CB1 antagonist) (119). CP-55940 disrupted sensory gating in the CA3 region of the hippocampus and entorhinal cortex and these effects were blocked by AM-251 showing that CB1 receptor agonists can disrupt sensory gating by altering neural oscillations relevant to perception and cognition.

The effects of cannabis extracts on nocturnal sleep, early morning performance, memory, and sleepiness were studied in in a double-blind, crossover study (120). Eight subjects were given placebo, 15 mg Δ^9^-THC, 5 mg Δ^9^-THC + 5 mg CBD, or 15 mg Δ^9^-THC + 15 mg CBD by oromucosal spray. There were no effects of 15 mg Δ^9^-THC on nocturnal sleep, although the next day, subjects reported increased sleepiness shortly after rising, and there were changes in mood and decreased latencies to early-morning sleep. The Δ^9^-THC + CBD drug combinations produced a decrease in slow wave sleep, and wakefulness was increased with the higher dose combination. The next morning, there were no changes in mood, sleepiness, fatigue, or performance with the lower dose combination, but with the higher dose combination, subjects reported increased sleepiness with fatigue and changes in mood. The subjects treated with Δ^9^-THC alone also reported changes in mood and several aspects of memory were impaired. For both Δ^9^-THC + CBD combination doses, there were no changes in performance on the memory tests, apart from a reduced reaction time in digit recall with the lower doses.

### Driving performance

The effect of CBD and Δ^9^-THC on driving performance was evaluated in a placebo-controlled, double-blind, randomized, cross-over clinical trial in 26 occasional cannabis users (121). Subjects inhaled vaporized Δ^9^-THC (13.75 mg), CBD (13.75 mg), Δ^9^-THC (13.75 mg) + CBD (13.75 mg) [high Δ^9^-THC/high CBD] or placebo. CBD had no effect on driving performance, but it was significantly impaired after taking either Δ^9^-THC or the high Δ^9^-THC/high CBD combination. In an earlier randomized, placebo-controlled, double-blind, cross-over study by this group (122), the effects of high Δ^9^-THC and high Δ^9^-THC/high CBD cannabis were assessed on simulated driving and cognitive performance in 14 subjects with a history of light cannabis use. Subjects inhaled 125 mg of THC-dominant (11% Δ^9^-THC; < 1% CBD), high Δ^9^-THC/high CBD (11% Δ^9^-THC, 11% CBD), or placebo (< 1% Δ^9^-THC/CBD) cannabis. High Δ^9^-THC alone or high Δ^9^-THC/high CBD increased lane weaving but had little effect on other driving performance measures. Confidence in driving ability did not vary with CBD content. These interventions also impaired performance on the Digit Symbol Substitution Task, Divided Attention Task and Paced Auditory Serial Addition Task, with impairment on the latter two tasks worse with the high Δ^9^-THC/high CBD combination than high Δ^9^-THC cannabis.

The clinical studies that have been conducted to explore the interplay between the CNS effects of Δ^9^-THC and CBD present a mixed picture with some reporting adverse consequences of Δ^9^-THC/CBD combinations, some beneficial consequences, but for the majority there was no interaction. The relatively minor beneficial effects of CBD that have been reported do not support the hypothesis that CBD can have a substantial positive benefit on the psychotomimetic effects of cannabis on its adverse influence on psychomotor and cognitive function.

### Abuse potential evaluation

In the driving performance study conducted by Arkell et al. (2019) (122), the subjective effects of inhaling 125 mg of THC-dominant (11% Δ^9^-THC; < 1% CBD), high Δ^9^-THC/high CBD (11% Δ^9^-THC, 11% CBD) or placebo (< 1% Δ^9^-THC/CBD) cannabis were determined in 14 subjects with a history of light cannabis use. The subjective effects of Δ^9^-THC, e.g. feeling “stoned” or sedated, were not influenced by CBD content.

Spindle et al. (2020) (123) examined the effects of oral or vaporized administration of CBD or CBD-dominant cannabis in a double-blind, placebo-controlled trial in 18 healthy adults with experience of cannabis use. Subjects self-administered oral CBD (100 mg), vaporized CBD (100 mg), vaporized CBD-dominant cannabis (100 mg CBD + 3.7 mg Δ^9^-THC) or placebo. The subjective effects of oral CBD did not separate from placebo. Vaporized CBD and CBD-dominant cannabis produced discriminable subjective effects, which were sometimes stronger in women, but these interventions did not produce cognitive/psychomotor impairment. The effects of CBD-dominant cannabis were generally higher than vaporized CBD. The effect of oral CBD (0, 200, 400, 800 mg) pretreatment on the reinforcing, subjective, cognitive and physiological effects of inhaled cannabis (zero Δ^9^-THC [placebo] or 5.30-5.80% Δ^9^-THC) was studied in a randomized, double-blind, within-subject trial involving 31 cannabis smokers (124). Under the zero CBD condition, Δ^9^-THC-containing cannabis was self-administered by significantly more subjects than placebo cannabis. Δ^9^-THC-containing cannabis produced time-dependent increases in ratings of ‘high’, ‘good effect’, ratings of the cannabis cigarette (e.g., strength, liking), and changes in heart rate relative to inactive cannabis. CBD alone did not produce psychoactive or cardiovascular effects, and it did not significantly alter any of the Δ^9^-THC-induced outcomes, demonstrating that CBD did not reduce the reinforcing, physiological, or positive subjective effects of cannabis. In agreement with these findings, a randomized, double-blind, placebo-controlled, crossover study (125) compared the acute effects of cannabis in adolescent and adult cannabis users (24 subjects/group) to evaluate whether co-administration of CBD could modulate the acute effects of Δ^9^-THC. Δ^9^-THC (8 mg) alone and the Δ^9^-THC/CBD combination (8 mg Δ^9^-THC + 24 mg CBD) both significantly increased the subjective measure of ‘feel drug effect’, impaired verbal episodic memory and increased psychotomimetic effects (PSI score) with no difference between adolescents and adults.

Not all studies have been negative. In a randomized, double-blind, crossover, placebo-controlled study in 18 subjects, Sainz-Cort et al. (2021) (126) evaluated the effect of CBD to reduce some of the psychotomimetic effects of Δ^9^-THC in cannabis users. Participants were administered cannabis extracts containing Δ^9^-THC, CBD, Δ^9^-THC/CBD, or placebo. CBD did not induce any psychotomimetic effects and it reduced some of the psychotomimetic effects of Δ^9^-THC.

Solowij et al. (2019) (127) conducted a randomized trial to examine the acute effects of Δ^9^-THC and CBD alone and in combination, administered by vaporization to frequent and infrequent cannabis users. Male subjects (34) inhaled vaporized CBD (400 mg); Δ^9^-THC (8 mg), high Δ^9^-THC/low CBD [Δ^9^-THC (8 mg) + CBD (4 mg)], high Δ^9^-THC/high CBD [Δ^9^-THC (8 mg) + high CBD (400 mg)] or placebo. CBD alone produced some sedative and intoxicating effects. High Δ^9^-THC/low CBD enhanced, while high Δ^9^-THC/high CBD reduced, the intoxicating effects of Δ^9^-THC. These effects were particularly prevalent in infrequent cannabis users and consistent across objective and subjective measures.

In a small, placebo-controlled, double-blind crossover study (128), 8 normal healthy volunteers (no prior experience of cannabis use required) were given Δ^9^-THC (0.5 mg/kg), CBD (1 mg/kg), Δ^9^-THC/CBD [Δ^9^-THC (0.5 mg/kg) + CBD (I mg/kg)] with placebo and diazepam (10 mg) as negative and positive controls, respectively. CBD blocked the cannabis-like effects and other subjective alterations induced by Δ^9^-THC (Addiction Research Center Inventory for Marihuana Effects, the Analogue Self-Rating Scale for Subjective Feelings and Scale of Bodily Symptoms). The authors concluded the CBD effect was not caused by a general blockade of Δ^9^-THC effects because no change in pulse-rate measurements was detected.

Neural correlates of reward anticipation were investigated in adolescents and adults after acute exposure to cannabis in a double-blind, placebo-controlled, randomized, crossover trial in a population of frequent cannabis users (129). Adolescents (24/group) and adults (23/group) completed the Monetary Incentive Delay task during functional magnetic resonance imaging (fMRI) after inhaling cannabis with Δ^9^-THC (0.107 mg/kg), Δ^9^-THC/CBD [Δ^9^-THC 0.107 mg/kg + CBD (0.320 mg/kg)] or placebo. Δ^9^-THC alone reduced reward anticipation activity in the right and left ventral striatum and right insula and the Δ^9^-THC/CBD combination reduced this signal in the right ventral striatum and right insula. There were no significant effects in the whole-brain analyses. No differences were found between the Δ^9^-THC and Δ^9^-THC/CBD interventions, or between the two age groups. The results indicate that CBD does not influence the Δ^9^-THC-induced suppression of the brain’s anticipatory reward response.

fMRI has also been employed in 15 male subjects who were infrequent cannabis users to assess whether Δ^9^-THC and CBD produced opposite effects on regional brain function, and if pretreatment with CBD prevented the acute psychotic symptoms induced by Δ^9^-THC (130). Relative to placebo, orally administered Δ^9^-THC (10 mg) and CBD (600 mg) produced opposite effects on activation in striatum during verbal recall, in hippocampus during the response inhibition task, in amygdala when they viewed fearful faces, in superior temporal cortex when they listened to speech, and in occipital cortex during visual processing. Using the PANSS rating scale in a further 6 subjects, intravenous pretreatment with CBD (5 mg) prevented the acute induction of psychotic symptoms produced by intravenous Δ^9^-THC (1.25 mg).

Clinical studies investigating whether CBD influences the positive reinforcing and negative psychotomimetic effects of cannabis has thrown out more conflicting results than other clinical and non-clinical research into the interaction between CBD and Δ^9^-THC. With no clear outcome from this area of research, further studies to elucidate whether CBD can reduce the abuse-related harms associated with Δ^9^-THC are warranted.

## Other safety concerns

One of the challenges when attempting to make an objective assessment of the safety risks posed by CNS-active drugs is sourcing information that is not open to bias, artifacts, or confounders. We are fortunate that Δ^9^-THC (Marinol™ and Syndros™), its close analogue nabilone (Cesamet™), CBD (Epidiolex™) and 1:1 Δ^9^-THC/CBD combination (Sativex™) have undergone rigorous non-clinical and clinical safety evaluation. In these studies, the compound and its purity are known, “blinding” of treatments and placebo controls have been applied.

The US Product Label for Marinol™ (dronabinol; synthetic Δ^9^-THC) describes the expected CNS effects of “highs” with elation and heightened awareness in both antiemetic (24%) and the lower-dose appetite stimulant clinical trials (8%) (3). As predicted by the non-clinical experiments, these effects are most pronounced in the first 2 weeks of treatment and then rapidly subside over the next few weeks. Adverse events linked to the intoxicating action of Δ^9^-THC were also reported by 3-10% of subjects in clinical trials. The drug did induce a withdrawal syndrome after discontinuation of 210 mg/day for 12-16 consecutive days that started with irritability, insomnia, restlessness progressing to “hot flashes,” sweating, rhinorrhea, loose stools, hiccoughs, and anorexia. The withdrawal symptoms gradually dissipated over the next 48 hr. The only other safety concern was use in pregnancy because of some adverse findings in the non-clinical reproductive toxicity studies. It should be noted, however, that these effects occurred at doses 15-20-fold higher than the highest clinical dose of Marinol™.

Cesamet™ (nabilone) is a more potent, synthetic analogue of THC. The Cesamet™ - US Product Label acknowledges its effects on the mental state are similar to those of cannabis. The adverse events in clinical trials showed evidence of euphoria (11% of subjects), sedation (drowsiness, 52% of subjects) and intoxication (vertigo and ataxia in 52% and 19% of subjects, respectively). There were no adverse events in long-term toxicity studies in monkeys or reproductive toxicity studies in rats. The one area where Cesamet™ differed from Marinol™ was the former can elevate supine and standing heart-rates and cause postural hypotension (5).

Epidiolex™ (CBD) is non-psychoactive and not a Controlled Drug and consistent with this profile, there are no reports in the product label to indicate euphoria, dissociation, intoxication or withdrawal (2). It is stated that Epidiolex™ produced sedation, but that adverse event occurred in patients with Lennox-Gastaut and Dravet Syndromes, not in subjects with tuberous sclerosis complex, suggesting it is related to the patient group, not the drug. The only safety concern is potential liver damage (elevated transaminases); however, a careful examination of the data revealed no evidence of this adverse event in subjects taking Epidiolex™ as monotherapy at doses as high as 10 mg/kg/day (131).

Several double-blind trials have been conducted with CBD, often to evaluate whether it modifies the CNS effects of Δ^9^-THC or cannabis. Babalonis et al. (2017) (132) studied the CNS effects of oral CBD (0, 200, 400 and 800 mg) in a group of healthy, frequent users of marijuana and found that in contrast to active marijuana that reliably produced abuse-related effects, CBD did not separate from placebo on key measures including “high”, “good drug effect”, “sedated”, “mellow”, and “marijuana good effects”. Similarly, Haney et al. (2016) (124) observed that oral CBD (0, 200, 400, 800 mg) produced no reinforcing (e.g. “high”, “drug liking”, “marijuana strength” and “marijuana street value”), subjective, cognitive, or physiological effects in healthy cannabis smokers. Morgan et al. (2018) (114) arrived at similar conclusions about the lack of reinforcing, intoxicating, and cognitive impairing effects of CBD when they studied vaped 16 mg of CBD in a group of marijuana users. The one study that claims to have observed intoxicating and cannabis-like effects in marijuana users was by Solowij et al. (2019) (127) who studied the effects of a 400 mg vaped dose of CBD in groups of frequent and infrequent marijuana users. While the results may be robust, they were obtained in a small group of subjects and the doses were not relevant either to the clinical or non-clinical use of CBD.

Sativex™ (plant derived 1:1 Δ^9^-THC/CBD) is taken as an oral mucosal spray. The Sativex™ - EU Summary of Product Characteristics (6) lists CNS adverse events with a frequency and spectrum that is similar with Marinol™ and Cesamet™; thus, there is evidence of “highs” (e.g. euphoria, and dissociation ≤10% of subjects), sedation (e.g. lethargy and somnolence ≤10% of subjects) and intoxication (e.g. disorientation, amnesia, memory impairment and impaired attention ≤10% of subjects). Safety pharmacology toxicity testing, including reproductive toxicity testing, produced no results to indicate a risk of harm to humans.

The findings obtained from clinical experience and placebo-controlled trials with Δ^9^-THC, synthetic CB1/CB2 agonists, CBD and Δ^9^-THC/CBD combinations broadly agree with the findings from the non-clinical pharmacology and abuse/dependence evaluation.

In addition to their abuse and dependence risks, various other potential harms are known to be associated with the use of cannabis and synthetic psychoactive cannabinoids. These factors including the link between cannabis use and psychosis, increased risk of testicular cancer, adverse neurodevelopmental and reproductive harms, and the role of cannabis use as a “gateway drug” leading to the use of alcohol, tobacco and highly addictive drugs like the opioids and cocaine have been comprehensively analyzed by the US National Academies of Sciences, Engineering, and Medicine in 2017 (133). They have also been extensively investigated by other research groups, e.g. the link between cannabis use and psychosis (134–136), increased risk of testicular cancer (137, 138), adverse neurodevelopmental and reproductive harms (139–141), and the role of cannabis use as a “gateway drug” (142–146). None of these factors is directly relevant to an analysis of the abuse and dependence risks posed by naturally occurring and synthetic cannabinoids. Nonetheless, they are important issues to be considered in decision-making on the legalization of cannabinoids for self-diagnosed medical or recreational use.

Any legislative decision to legalize cannabis, which includes Δ^9^-THC and related naturally-occurring, psychoactive cannabinoids, will incorporate a mandatory minimum age for legal access to and use of these substances. It has long been believed that the younger the age of exposure to the use of legal or illicit substances of abuse, the greater the risk of developing a related substance use disorder. Volkow et al. (2021) (147) investigated the risk of transitioning from substance use (tobacco, alcohol, cannabis, cocaine, methamphetamine, and heroin, opioids, stimulants, and tranquilizers) to substance use disorder in cohorts of adolescents (12-17 years) and young adults (18-25 years). The analysis revealed that the probability of developing cannabis use disorder increased substantially if exposure to cannabis occurred in adolescence. The increased risk was not observed with alcohol or cigarette use. This finding should prompt an enhanced level of vigilance to restrict the access of minors to cannabis and its psychoactive cannabinoids.

## Summary/conclusions

This review has attempted to collate evidence from well designed, rigorously controlled and conducted, non-clinical and clinical sources to evaluate the safety risks associated with the psychoactive and non-psychoactive cannabinoids present in cannabis. To provide context to the results, we also evaluated a wide range of synthetic cannabinoids, mostly of the psychoactive class.

The following conclusions can be drawn:

1. Δ^9^-THC and the other psychoactive cannabinoids in cannabis have moderate reinforcing effects when compared against the full spectrum of substances of abuse.
2. Although the psychoactive cannabinoids induce tolerance, in contrast to the opiates, benzodiazepines and barbiturates, it does not result in the development of severe physical dependence.
3. Discontinuation after repeated exposure to Δ^9^-THC and the other psychoactive cannabinoids in cannabis can result in a moderately severe withdrawal syndrome which lasts from 2 to 6 days.
4. The evidence overwhelmingly shows that non-psychoactive cannabinoids do not produce intoxicating, cognitive or rewarding properties in animals or humans.
5. The area where the greatest discordance exists between studies is whether CBD influences the CNS effects of Δ^9^-THC or cannabis. Although most investigations have shown that CBD does not attenuate any of the CNS-effects of Δ^9^-THC and other psychoactive cannabinoids, there is sufficient disagreement to warrant further research in this area.

Using the findings to predict the level of risk and harm to users, their family and social groups and the public, our assessment is cannabis carries a moderate level of risk. While the risks and harms are substantially lower than those of many illegal and legal substances of abuse, including tobacco and alcohol, they are far from negligible. One of the lessons learned from the opioid abuse crisis is relaxation of prescribing rules to allow greater patient access to these drugs greatly magnified the level of public risk. By the same logic, legalization of cannabis use would in all probability result in an increase in the prevalence of cannabis use disorder, particularly amongst the young.

The potent synthetic CB1/CB2 agonists are a very different animal. They are substantially more reinforcing than Δ^9^-THC, highly intoxicating, and their frequent use produces rapid tolerance with the potential for dose-escalation. Discontinuation produces greater adverse withdrawal effects. These synthetic cannabinoids pose significant risks for abuse and harm and should not be legalized.

## Author contributions

DH: Conceptualization, Investigation, Writing – original draft. JG: Conceptualization, Investigation, Writing – original draft. SS: Conceptualization, Investigation, Writing – original draft.
